# DNA Capture and Enrichment: A Culture-Independent Approach for Characterizing the Genomic Diversity of Pathogenic *Leptospira* Species

**DOI:** 10.3390/microorganisms11051282

**Published:** 2023-05-14

**Authors:** Nathan E. Stone, Ryelan F. McDonough, Camila Hamond, Karen LeCount, Joseph D. Busch, Katherine L. Dirsmith, Sarai Rivera-Garcia, Fred Soltero, Laura M. Arnold, Zachary Weiner, Renee L. Galloway, Linda K. Schlater, Jarlath E. Nally, Jason W. Sahl, David M. Wagner

**Affiliations:** 1 The Pathogen and Microbiome Institute, Northern Arizona University, Flagstaff, AZ 86011, USA; 2 National Veterinary Services Laboratories, Animal and Plant Health Inspection Service (APHIS), U.S. Department of Agriculture, Ames, IA 50010, USA; 3 Veterinary Services, Animal and Plant Health Inspection Service, U.S. Department of Agriculture, San Juan, PR 00918, USA; 4 Veterinary Diagnostic Laboratory, Department of Veterinary Science, University of Kentucky, Lexington, KY 40511, USA; 5 Bacterial Special Pathogens Branch, Centers for Disease Control and Prevention, Atlanta, GA 30333, USA; 6 Infectious Bacterial Diseases Research Unit, National Animal Disease Center, Agricultural Research Service, United States Department of Agriculture, Ames, IA 50010, USA

**Keywords:** leptospirosis, *Leptospira*, genome sequencing, DNA enrichment

## Abstract

Because they are difficult to culture, obtaining genomic information from *Leptospira* spp. is challenging, hindering the overall understanding of leptospirosis. We designed and validated a culture-independent DNA capture and enrichment system for obtaining *Leptospira* genomic information from complex human and animal samples. It can be utilized with a variety of complex sample types and diverse species as it was designed using the pan-genome of all known pathogenic *Leptospira* spp. This system significantly increases the proportion of *Leptospira* DNA contained within DNA extracts obtained from complex samples, oftentimes reaching >95% even when some estimated starting proportions were <1%. Sequencing enriched extracts results in genomic coverage similar to sequenced isolates, thereby enabling enriched complex extracts to be analyzed together with whole genome sequences from isolates, which facilitates robust species identification and high-resolution genotyping. The system is flexible and can be readily updated when new genomic information becomes available. Implementation of this DNA capture and enrichment system will improve efforts to obtain genomic data from unculturable *Leptospira*-positive human and animal samples. This, in turn, will lead to a better understanding of the overall genomic diversity and gene content of *Leptospira* spp. that cause leptospirosis, aiding epidemiology and the development of improved diagnostics and vaccines.

## 1. Introduction

Leptospirosis is the most widespread bacterial zoonosis globally and is capable of infecting many different mammalian species [1,2,3]. More than 1 million human cases of leptospirosis are estimated to occur annually with a fatality rate of ~6% [4]. However, these statistics are likely gross underestimations as leptospirosis diagnostics can be unreliable or unavailable in many countries. Indeed, it is frequently misdiagnosed as dengue, malaria, or other acute febrile tropical diseases [5]. The definitive diagnostic for leptospirosis is a positive culture, which is difficult to obtain due to slow growth rates/long incubation times, fastidious growth requirements that can differ among species/serovars, bacterial contamination, and the requirement for sample collection prior to the initiation of antibiotic treatment [1]. Although leptospires are distributed worldwide, human leptospirosis mainly affects urban and rural low-income communities in tropical regions [2,4,6]. Recently, human leptospirosis has been increasingly reported in industrialized countries and temperate regions, possibly due to rising ambient temperatures and humidity [7].

*Leptospira* also causes disease in a wide variety of domestic animals, thus, leptospirosis is also of great importance to veterinary medicine [8]. Domestic and wild animals are essential to the transmission cycle of leptospirosis and are an important source of human infections. They often act as maintenance hosts wherein infectious leptospires colonize the kidneys and renal tubules and then are shed through urine [1,9]. Chronic infection with intermittent shedding in some bovines can occur for >12 months [10] leading to significant economic losses [11], and rats (*Rattus rattus* and *R. norvegicus*) are commonly infected asymptomatically and leptospires can transmit among them [12]. Urine-contaminated soil and water lead to transmission to humans and other animals [9]. As a result, humans that are exposed to these sources are at increased risk for leptospirosis, including agriculture workers, participants in water sports, and those living in resource-poor conditions [13]. Risk to humans is increased during heavy rains and flooding events and, as climactic conditions change and these events are becoming more frequent, the impacts become more devastating [14,15,16].

Existing diagnostic tools for human and animal leptospirosis are suboptimal with regard to sensitivity, specificity, useability, and availability [5,17]. *Leptospira* is a diverse genus and, as such, pathogenic leptospires of varying serogroups may evade detection via the microscopic agglutination test (MAT), the “Gold Standard” and most widely used serological diagnostic test for leptospirosis, which was developed over a century ago [17]. Diagnosis by MAT relies on two separate samples taken during both the acute and convalescent stages of the disease, requires a skilled laboratorian and a diverse set of reference isolates to execute, is not widely available, and yields results that are often inconclusive [18]. In many cases, serological results from MAT are confusing or conflicting when paired with genotyping results from PCR [19]. Furthermore, MAT is only capable of detecting exposure from serogroups that have been included in the assay panel, and MAT panels typically include only the most common serogroups containing representative serovars (often no more than 5–7) [20]; there are more than 20 leptospirosis serogroups with >300 serovars currently described [17]. Furthermore, serogroups/serovars and *Leptospira* species vary in presence and abundance in different geographic regions [12,21], so unexpected/novel/undescribed lineages could easily go undetected, leading to a false negative leptospirosis diagnosis [22]. Compared to MAT, PCR may be more sensitive in certain stages of the disease progression [23] and provide a longer-term “detection window” for the diagnosis of chronic leptospirosis because leptospires can be shed for months in the urine of chronic carriers [10].

Current PCR-based leptospirosis diagnostics (e.g., those targeting *lipL32* [24] or *secY* [25]) are valuable detection and coarse genotyping tools but provide only limited species/strain level detection and discriminatory information on their own, and can fail to amplify in some diverse strains [26]. Therefore, a thorough understanding of the infectious lineages/species/serogroups/serovars circulating in all regions of the world is critical to improving the functionality of these leptospirosis diagnostics. *Leptospira* is a highly diverse genus with genomes larger than other spirochetes with high genomic variability [27], potentially explaining its ability to survive in a variety of hosts (humans and domestic and wild animals), environmental conditions (soil and water), and climates (tropical, arid, others) [28]. However, genomic information is severely lacking for *Leptospira* spp. due to the challenge of obtaining purified isolates and, without this information, the development of improved diagnostics and vaccines is thereby impeded [1,13]. Genomic information is also critical to epidemiological investigations, which in turn aid in disease and transmission mitigation efforts. Genomic sequences are typically generated from purified cultures, thus pathogens that are easily culturable in the lab have large databases of genomic information to facilitate these developments (e.g., *Mycobacterium tuberculosis*, *Escherichia coli*, *Salmonella* spp.). However, because leptospires are difficult and oftentimes impossible to culture, a comprehensive database of *Leptospira* genomic information is lacking, and this genomic shortcoming needs to be remedied to facilitate the advancement of leptospirosis research to improve public health outcomes for humans and animals.

To address this need and supplement and improve collective public *Leptospira* genomic resources, we describe here the design and validation of a pan-pathogenic *Leptospira* DNA capture and enrichment system that can be used to obtain genomic information from unculturable leptospirosis clinical and animal samples, including frozen and archived samples and those that have been collected after treatment with antibiotics [29].

## 2. Materials and Methods

### 2.1. DNA Capture and Enrichment Probe Design

The design of DNA capture probes is a scalable and iterative process wherein new probes can be added to an existing system as new and novel genomes become available; similarly, unwanted probes (e.g., those determined to be hybridizing to non-target sequences) can also be removed. The original design for our pan-pathogenic *Leptospira* DNA capture and enrichment system (v1) was based on 482 publicly available *Leptospira* spp. genomes representing nine pathogenic species (Table S1) and contained 212,311 RNA probes. We subsequently updated that original design (v2) to include novel *Leptospira* genomic content from *L. sanjuanensis* [30] and additional genomes from known species that became available in public databases throughout this study; it now contains 297,795 probes based upon 502 genomes representing 13 pathogenic *Leptospira* spp. from the pathogenic clade P1 (Table S2). The general design process is explained in detail elsewhere where we describe a similar enrichment system for *Francisella* spp. [29], but in brief, the process consisted of (1) bioinformatically “slicing” the coding sequences into 120 nucleotide (nt) fragments and designing complimentary RNA probes with 2x tiling (probes overlap by 60 nts) to maximize coverage; (2) removing probes that were only conserved in a single genome because these sequences may represent contamination; and (3) removing probes that capture highly conserved regions (e.g., rRNA genes) as well as those that show homology with non-target bacteria to minimize hybridization and capture of unwanted sequences. Regions with low GC content are difficult to hybridize [29]; to compensate for this difficulty, additional probes for these regions were added to the design. The final probe set was ordered from Agilent (Agilent SureSelect catalog# 5191-6920, Santa Clara, CA, USA).

### 2.2. Samples Utilized for DNA Capture and Enrichment

All samples used in this study and the analyses applied to each are summarized in Table 1. Throughout the text, we use the term “complex sample” to refer to DNA extracts that contain nucleic acids from multiple species, including hosts, bacteria, and/or other environmental organisms. The term is used to broadly describe any DNA extract that is not derived from an isolated bacterial culture. The samples were divided into a validation set and an unknown set. As described below, comparative isolates were available for all of the eight complex samples in the validation set; no comparative isolates were available for the five complex samples in the unknown set.

### 2.3. Validation Set

To validate the DNA capture and enrichment system we generated two “mock” samples. These were two separate DNA extracts of human urine negative for *Leptospira* but positive for *E. coli* that varied in molecular weight: Mock1 was highly fragmented [~75 base pairs (bp)], whereas most fragments for Mock2 were ~1500 bp. To mimic a low-level *Leptospira* infection, both extracts were spiked with ~2.62 × 10^−6^ ng/µL of gDNA [concentration based upon Qubit measurements (see method below)] from *L. interrogans* serovar Copenhageni strain Fiocruz L1-130. As a point of reference, *lipL32* qPCR Ct values for the spiked Mock1 and Mock2 samples, which are used as a proxy for bacterial load, were 37.04 and 36.17, respectively. Because our gDNA stock of *L. interrogans* serovar Copenhageni strain Fiocruz L1-130 was purchased from a commercial distributor (ATCC, Manassas, VA, USA, catalog# BAA-1198D-5) and is derived from a laboratory-maintained bacterial stock that likely continues to accumulate mutations over time, we generated a whole genome sequence for the gDNA stock to serve as a more precise comparison to the enriched sequences that were generated from the mock samples, rather than relying solely on the previously published genome assembly for this strain (GenBank accession# GCA_000007685.1).

The validation set also included five enriched complex bovine samples. Two were separate urine voids obtained from the same dairy bovine (designated MN900) from which an *L. borgpetersenii* serovar Tarassovi isolate had recently been obtained and sequenced to completion as part of an earlier study [31]. One of these urine voids, Void1 12/9, was the source of that isolate (collected on 9 December 2020 and also designated MN900) [31] and, as such, was positive via *lipL32* PCR (Ct = 22.08); the other, Void2 12/9, was *lipL32* PCR negative and, thus, was used here as a negative control. The three other bovine samples were urine voids (DCP009, DCP017, and DCP041) from three separate bovines in Puerto Rico from which isolates (also designated DCP009, DCP017, and DCP041) had been previously obtained and sequenced to completion as part of an earlier study [32]. The urine voids used to generate the enrichments for DCP009, DCP017, and DCP041 were collected on 16 December 2020, 13 January 2021, and 19 May 2021, respectively, whereas the urine voids used to obtain the cultured isolates were collected on 10 February 2021 for isolates DCP009 and DCP017, and 12 August 2021 for isolate DCP041.

Finally, to assess the ability of this DNA capture and enrichment system to enrich pathogenic *Leptospira* species from a complex environmental sample, our validation set also included soil sample 16^S^-27 from Puerto Rico, which is the same soil sample that yielded isolates (LGVF01 and LGVF02) of the recently described *L. sanjuanensis* [30]. In addition to the pathogenic *L. sanjuanensis* isolates, soil sample 16^S^-27 also produced multiple saprophytic *Leptospira* spp. isolates and was suspected to contain at least two additional pathogenic *Leptospira* lineages based on sequence analysis of *lipL32* and *secY* amplicons [26].

### 2.4. Unknown Set

The unknown set contained two complex samples (blood and urine) obtained from two different humans and three complex samples (urine) obtained from three different bovines. Human samples were collected under CDC IRB protocol# 7201, whereas cattle samples were collected as part of case investigations or for disease surveillance. The two human samples, PCRpos02 and PCRpos05, originated from Puerto Rico and were positive via *lipL32* PCR (Ct = 28.03 and 31.99, respectively) but did not yield isolates. PCRpos02 was a blood sample and was suspected to contain *L. interrogans* based on a species-specific qPCR panel [33]. PCRpos05 was a urine sample and was suspected to be infected with *L. kirschneri* based on the same species-specific qPCR assays. Two of the bovine samples, KY74 from an adult and KYcalf from a calf, were collected in Kentucky in December 2020 and were both *lipL32* PCR positive. The third bovine sample, WI878, was collected in Wisconsin in April 2021 and was *lipL32* and *secY* PCR negative but FAT (fluorescent antibody test) positive using *Leptospira* fluorescent antibody conjugate (National Veterinary Services Laboratories, APHIS, USDA, Ames, IA, USA) produced with multivalent high-titer rabbit anti-sera to serogroups Canicola, Grippotyphosa, Hardjo, Icterohaemorrhagiae, and Pomona. WI878 also had a weak band for 16S PCR [34] that presented low identity to *L. interrogans* when sequenced. Species identification was undetermined for these three bovine samples and isolates were not obtained.

### 2.5. DNA Extraction

Because DNA was acquired from various collaborators and multiple laboratories, extraction methods varied among samples. Human urine samples used to generate the mock samples were extracted using Norgen Urine DNA Isolation kits (Norgen, Thorold, ON, Canada). For the MN900 urine voids (Void1 12/9 and Void2 12/9) DNA was extracted from urine pellets using DNeasy Blood and Tissue kits (Qiagen, Valencia, CA, USA) according to the manufacturer’s recommendations, except the buffer AL incubation step occurred at 80 °C for 1 h. DNA was extracted from soil sample 16^S^-27 using a PowerSoil kit (Qiagen, Valencia, CA, USA), as previously described [26]. DNAs from the remaining human and bovine samples were provided by the CDC and USDA, respectively, and were extracted using Maxwell kits (Promega Corporation, Madison, WI, USA) [32].

### 2.6. DNA Capture and Enrichment

Prior to DNA capture and enrichment, all DNA extracts were assessed for quality and quantity by Qubit BR or HS dsDNA kits (Thermo Fisher Scientific, Waltham, MA, USA) and Fragment Analyzer genomic DNA analysis kits (Agilent Technologies, Santa Clara, CA, USA) and subjected to *lipL32* PCR as previously described [24,26,31,35,36]. DNAs were then diluted to ≤4 ng/µL for input into the capture and enrichment process, sonicated to obtain optimal fragment size for the capture step (~250 bp), and then uniquely indexed libraries were prepared for each sample according to the SureSelect XT-low input Target Enrichment System protocol (Agilent Technologies, Santa Clara, CA, USA). Certain samples were pooled prior to enrichment whereas others were not (Table 1: Pooling), and a slow hybridization method was implemented to prevent the dissociation of probes from AT-rich regions, with ~1000–2000 ng of each library hybridized at 65 °C for 16–24 h. Libraries were then subjected to one or two rounds of DNA capture and enrichment. All these methods are described in more detail elsewhere [29].

We confirmed the presence or absence of *Leptospira* DNA in each sample library prior to enrichment, and also assessed the increase in the proportion of *Leptospira* DNA after each round of enrichment, using a novel qPCR assay (“LeptoBait”) designed to target a conserved probe in the capture and enrichment system; this assay utilizes primer pair LeptoBaitF1, 5′TTACTCAAAGGATTTAAACGTCC and LeptoBaitR1, 5′CTCTGCAACGAACTTCCC. The assay was performed on the sequence-ready libraries prior to enrichment using ~20 ng of input DNA and after each round of enrichment using ~1 ng of input DNA. We utilized a 5-fold serial dilution of our *L. interrogans* gDNA control (strain Fiocruz L1-130) to generate a standard curve (starting concentration was 2.62 ng/µL) and used this standard curve to estimate the concentration of *Leptospira* DNA in our starting and enriched libraries. PCRs were carried out in 10 µL volumes containing the following reagents (given in final concentrations): 1 µL of diluted DNA template, 1x SYBR^®^ Green Universal master mix (Applied Biosystems, Foster City, CA, USA), and 0.4 µM of each primer. The assay was run on an Applied Biosystems 7500 Fast Real-Time PCR System with SDS 7500 software v2.0.6 under the following conditions: 50 °C for 2 min, 95 °C for 10 min, and 40 cycles of 95 °C for 15 s and 58 °C for 1 min; positive and non-template controls were included on all runs. Although an enrichment library was successfully generated for samples 16^S^-27 and WI878 (based upon Qubit and Fragment Analyzer), we were unable to detect *Leptospira* DNA using our LeptoBait qPCR following the enrichment process. Regardless, we proceeded with Illumina sequencing of these samples to explore the possibility that *Leptospira* DNA was being captured but perhaps fell below our limit of detection with this qPCR assay. For certain samples (Table 1: Post Capture % Increase), we quantified the level of *Leptospira* in the pre-enriched library in terms of the proportion of total *Leptospira* DNA present in a sample by dividing the estimated concentration of *Leptospira* DNA (based upon LeptoBait qPCR) by the concentration of input DNA in that qPCR reaction, which was estimated by Qubit HS and BR dsDNA kits, as described above.

### 2.7. DNA Sequencing

Prior to sequencing, the final libraries from samples subjected to DNA capture and enrichment were quantified by qPCR using an Applied Biosystems QuantStudio 7 Flex Real-Time PCR System and the KAPA SYBR FAST ROX Low qPCR Master Mix for Illumina platforms (Illumina, San Diego, CA, USA, catalog# KK4873) and also assessed by Fragment Analyzer DNF-374 kits (Agilent Technologies, Santa Clara, CA, USA). Paired-end sequences were obtained on Illumina sequencers (MiSeq, MiniSeq, and NextSeq) using various kits (Mini-Seq Mid Output Kit [300 cycles], MiSeq Reagent Kit v2 [300 and 500 cycles], and Next-Seq 500/550 High Output KT v2.5 [300 cycles]) and standard Illumina procedures.

Genomic DNA from an isolate of *L. interrogans* serovar Copenhageni strain Fiocruz L1-130 was purchased from ATCC and assessed for quality and quantity on a 0.7% agarose gel using λ DNA-HindIII Digest (New England Biolabs, Ipswich, MA, USA). Whole genome sequencing library construction was performed on it using the KAPA Hyper Prep Kit for Illumina NGS platforms per the manufacturer’s protocol with double-sided size-selection performed after sonication (KAPA Biosystems, Woburn, MA, USA, catalog# KK8504). Adapters and 8 bp index oligos purchased from IDT (Integrated DNA Technologies, San Diego, CA, USA), based on previous work by Kozarewa and Turner [37], were used in place of those supplied in the KAPA Preparation kit. The final library was quantified, assessed for quality, and sequenced as described above for the enriched libraries on an Illumina MiSeq using the 500-cycle v2 kit with the standard Illumina procedure. Whole genome sequences using both Illumina and Nanopore reads were generated for isolates MN900, DCP009, DCP017, and DCP041 (BioSamples SAMN22138155, SAMN24659831, SAMN24659830, and SAMN24659832) by USDA-APHIS in Ames, IA, USA as part of other studies [31,32].

### 2.8. Bioinformatic Methods

The overall goals of the bioinformatic analyses described here were to quantify the genomic coverage obtained via DNA capture and enrichment followed by sequencing, and to characterize the quality and coverage of the enriched genomes in comparison to genomes obtained from cultured isolates. We also sought to understand the potential impact of pooling uniquely indexed sample libraries prior to enrichment on genomic quality and coverage, a strategy aimed at reducing costs. Furthermore, we assessed potential differences in genomic coverage among identical samples subjected to one versus two rounds of enrichment to elucidate what may be gained and/or lost during subsequent rounds of DNA capture and enrichment. Then, finally, we quantified genomic breadth of coverage at decreasing sampling depths (from >90x to <20x) to determine the optimal sequencing depth to target for enriched samples, which is an important consideration because enriched genomes appear to be subject to more uneven coverage (i.e., “peaks and valleys”) than traditional whole genome sequences. For all comparative analyses (pooled vs. non-pooled [Table 1: Pooling], one round of enrichment vs. two rounds [Table 1: Number of Enrichments], and sequencing depth [Table 1: Avg. Seq. Depth]), we normalized read counts.

### 2.9. Subsampling of Reads

An iterative subsampling approach was used to normalize paired Illumina read counts across samples. This was accomplished using seqtk v1.3 (https://github.com/lh3/seqtk; accessed on 1 May 2022) and a custom Python script (https://gist.github.com/jasonsahl/1281a2ae7f10382c773fec9bf7906d0c; accessed on 1 May 2022) that generated 100 random starting seeds, one to be used for each subsampling iteration. Subsampled reads for each of the 100 iterations were then mapped to a designated reference genome (Table 2) with minimap2 v2.22 [38] and the percent breadth of coverage ≥3x was calculated using a Samtools wrapper script (https://gist.github.com/jasonsahl/b5d56c16b04f7cc3bd3c32e22922125f; accessed on 1 May 2022) as previously described [24]; breadth and depth of coverage values were then averaged across all 100 iterations and those averaged values were used for all comparisons. Paired reads were either 150 bp or 250 bp in length depending on the sequencing kit used. As such, when making comparisons among samples with unequal read lengths, 250 bp reads were trimmed to 150 bp with Trimmomatic v0.39 [39] prior to subsampling.

### 2.10. Read Classification

To estimate the percentage of *Leptospira* reads in the enriched sequences, reads were mapped against the standard Kraken database with Kraken v2.1.2 [40]. During this process reads that are represented in the database are classified according to their taxonomic identity (total classified reads), whereas those that do not have a taxonomic representative in the database are undetermined (typically a minor fraction). The percentage of *Leptospira* reads was calculated by dividing the number of reads that were classified as *Leptospira* by the total number of reads per sample.

### 2.11. De Novo Assembly of Sequencing Reads

Sequencing reads were assembled using meta-SPAdes v3.13.0 [41] with default settings using all enriched reads and, separately, using only reads that were classified as *Leptospira* with Kraken2 (from above). For the latter, *Leptospira* reads were parsed from the FASTQ files. In addition to the enriched sequences, we also generated several assemblies to be used as genomic references. These assembled references were designated as RedPanda1_assembly.fasta and L1-130_assembly.fasta and were generated from *L. kirschneri* strain RedPanda1 (GenBank BioSample SAMN22327426) and our gDNA stock of *L. interrogans* serovar Copenhageni strain Fiocruz L1-130 (ATCC catalog# BAA-1198D-5), respectively (Table 2).

### 2.12. Hybrid Assemblies

We generated hybrid assemblies for isolates MN900, DCP009, DCP017, and DCP041 using Illumina and MinION raw sequencing reads (BioSamples SAMN22138155, SAMN24659831, SAMN24659830, and SAMN24659832). Illumina reads were trimmed with bbduk.sh v38.86 (https://sourceforge.net/projects/bbmap/; accessed on 1 May 2022), MinION reads were trimmed with Porechop v0.2.4 (https://github.com/rrwick/Porechop; accessed on 1 May 2022), and a hybrid assembly was created with Unicycler v0.4.8 (14) using default settings. The final assembly was polished using Pilon v1.23 (15) until no more corrections could be made. Assemblies were designated MN900_closed.fasta, DCP009_closed.fasta, DCP017_closed.fasta, and DCP041_closed.fasta (Table 2).

### 2.13. Species Identification and Determination of Mixtures

To determine the *Leptospira* spp. present in each sample we extracted ≤1383 bp of the *secY* gene [1383 bp = the complete coding sequence (CDS)] from the metagenome assembly using BLASTN [42] and a custom python script (https://gist.github.com/jasonsahl/2a232947a3578283f54c; accessed on 1 May 2022). To query for mixtures, we used minimap2 to align reads to *secY* (GenBank accession# MH059525.1), which generated a BAM file of the read pileup that was then visualized in Tablet [43]. Extracted consensus sequences (for all samples) and representatives of each unique sequence observed in the read pileups (for mixtures only) were then subjected to NCBI blastn (https://blast.ncbi.nlm.nih.gov/; accessed on 1 May 2022) to determine species.

### 2.14. Read Mapping and Calculations of Breadth and Depth of Coverage

Reads generated from enriched complex samples were aligned against appropriate reference genomes (Table 2) with minimap2 v2.22 and Samtools as described above and elsewhere [29].

### 2.15. Phylogenetic Comparisons between Isolates and Enrichments in the Validation Set

Single nucleotide polymorphisms (SNPs) were identified from raw enrichment sequence data, genome assemblies generated from isolates obtained from the same complex sample, and publicly available genome assemblies (GenBank accession numbers provided in figures). For raw sequence data, reads were aligned against reference genomes using minimap2 v2.22 [38] and calling SNPs from the BAM file with GATK v4.2.2 [44] using a depth of coverage ≥10x and a read proportion of 0.9. Genome assemblies were aligned against reference genomes with Nucmer v3.1 [45] and SNPs were called with NASP v1.2.1 [46]. Maximum likelihood phylogenies were then inferred on the concatenated SNP alignments using IQ-TREE v2.2.0.3 with the “-fast” option, default parameters [47], and the integrated ModelFinder method [48]. This analysis was not conducted for paired isolate/enrichment sample DCP017 because it was determined that this sample contained two infecting pathogenic *Leptospira* species in relatively equal proportions.

### 2.16. Direct Whole-Genome Comparisons between Isolates and Enrichments in the Validation Set

We also used NASP to directly compare closed isolate genomes to their enriched counterparts. We did these additional analyses because the phylogenetic analyses described above compare regions of the genome that are shared among all samples in the phylogeny, and thus, some overall genomic content is excluded when more diverse strains are included. By comparing the enriched genomes directly to their paired isolate genomes only, more genomic content is shared among these samples, which provides a more comprehensive understanding of the power and limitations of DNA capture and enrichment to make genomic-level epidemiological connections among very closely related isolates. For example, we sought to understand the conclusions that could be made when observing just a few SNP differences among genomes (derived from isolates and/or enrichments). We wanted to determine if these SNPs were robust, or if they could be the result of PCR error during the enrichment process, sequencing errors, and/or artifacts of the genomic analyses. To assess these possibilities, we leveraged our validation set of four samples from which we had paired enriched genomes and isolates that were sequenced to completion (Table 1 and Table 2).

### 2.17. Characterization and Phylogenetic Analysis of Enriched Genomes in the Unknown Set

For all five samples in this set, we determined the proportion of *Leptospira* DNA in the sample post-enrichment using Kraken2 and assigned species ID based upon *secY*. Enriched reads were then mapped against an appropriate reference genome (Table 2) to assess genomic coverage obtained during enrichment.

Samples PCRpos02, PCRpos05, and KY74 were subjected to whole genome phylogenetic analysis using the methods described above. PCRpos02 was analyzed among a diverse set of *L. interrogans* genomes using the L1-130_complete genome (GenBank accession# GCA_000007685.1) as the reference, whereas PCRpos05 was analyzed among a diverse set of available *L. kirschneri* genomes with our assembly of *L. kirschneri* strain RedPanda1 (RedPanda1_assembly.fasta) as the reference. Finally, KY74 was analyzed among a comprehensive set of *L. borgpetersenii* genomes using our assembly of MN900 (MN900_closed.fasta) as the reference. This analysis was not conducted for KYcalf due to the relatively equal mixture of two infecting pathogenic *Leptospira* species. The accession numbers for publicly available assemblies downloaded from GenBank are included in all phylogenies.

### 2.18. Pooling

Percent *Leptospira* reads, average breadth of coverage (minimum 3x), and average depth of coverage were calculated on subsampled reads (0.5–1 million) and plotted in GraphPad Prism v9.0.0 and evaluated using a paired *t*-test to assess statistical significance among sequencing results obtained for identical independently extracted sample libraries that were either pooled together in equimolar amounts prior to enrichment or enriched without pooling. Four sample libraries were used for this analysis (Table 2) and were analyzed as independent comparisons to illustrate the general trends, but also as a group to apply statistical support to the observed trends; *p* values < 0.05 were considered significant.

### 2.19. One vs. Two Rounds of Enrichment

Percent *Leptospira* reads, average breadth of coverage (minimum 3x), and average depth of coverage were calculated on subsampled reads (1–2 million) and plotted in GraphPad Prism v9.0.0 and evaluated using a paired *t*-test to assess statistical significance among results obtained for identical sample libraries subjected to one round of DNA capture and enrichment versus two rounds. Five samples that were sequenced after both one and two rounds of enrichment were used for this analysis (Table 2) and were analyzed as independent comparisons to illustrate general trends, but also grouped to apply statistical support to the observed trends; *p* values < 0.05 were considered significant.

### 2.20. Depth of Coverage

We subsampled 2.5 million, 2 million, 1.5 million, 1 million, and 0.5 million reads per sample and plotted the results in GraphPad Prism v9.0.0 and evaluated them using one-way ANOVA with Tukey’s correction for multiple comparisons to assess statistical significance among sampling depths; *p* values < 0.05 were considered significant. Pairwise comparisons for breadth and depth of coverage were conducted amongst all sampling depths.

### 2.21. De Novo Assembly Size

To assess the association of enriched genomes to de novo assembly using all sequencing reads, we plotted assembly size as a function of percent *Leptospira* DNA in the final enriched libraries.

### 2.22. Leptospira DNA Capture Probes Version 1 vs. Version 2

To ensure samples enriched with v1 and/or v2 DNA capture probes could be compared interchangeably, we enriched sample Void1 12/9 with both versions of the probe set and compared the breadth and depth of coverage between both versions separately, and also by combining the reads generated from both. We subsampled 1 million reads for each comparison and plotted the results in GraphPad Prism v9.0.0 and evaluated them using one-way ANOVA with Tukey’s correction for multiple comparisons to assess statistical significance among probe sets; *p* values < 0.05 were considered significant. By combining reads from both versions, we also assessed if the different designs were capturing different regions of the genome. For example, if v1 and v2 both obtained 90% breadth of coverage, but the combined coverage was 95%, it would indicate the v1 and v2 were capturing slightly different genomic targets. The version of the DNA capture probes used for each sample enrichment is indicated in Table 2.

## 3. Results

### 3.1. Validation Set

#### 3.1.1. Species Identification and Determination of Mixtures

The full-length *secY* gene (1383 bp) was extracted from the assemblies for all enrichment iterations for samples Mock1, Void1 12/9, DCP009, DCP017, and DCP041; whereas 866 bp and 1180 bp portions were extracted from Mock2-v1-R2 and Mock2-v1-R2-P, respectively; and an 890 bp portion from the enrichment of soil sample 16^s^-27-v1-R2; the reduced size was likely caused by incomplete capture of the *secY* gene for certain samples during enrichment. Species identifications were *L. interrogans* for Mock1 and Mock2; *L. borgpetersenii* for Void1 12/9, DCP009, and DCP041; *L. santarosai* for DCP017-v2-R1, but *L. borgpetersenii* for DCP017-v2-R2; and *Leptospira* spp. for 16^s^-27 (Table 2). Sequence identity of 100% to each species was observed with one exception; for sample 16^s^-27, *L. kmetyi* was the closest match with 92.47% sequence identity, a finding corroborating the results of our previous analysis of this sample [26]. Both *L. santarosai* and *L. borgpetersenii* reads were observed in the read pileup of the *secY* gene for sample DCP017 in relatively equal proportions, an observation corroborated by the species assignment of the consensus *secY* sequence to both *L. santarosai* and *L. borgpetersenii* for this sample (Table 2). A mixture of *L. borgpetersenii* and *L. santarosai* reads were also observed in enriched reads from sample DCP009, but the *L. santarosai* reads were only a minor fraction.

#### 3.1.2. Enrichment Results

**Figure 1:**
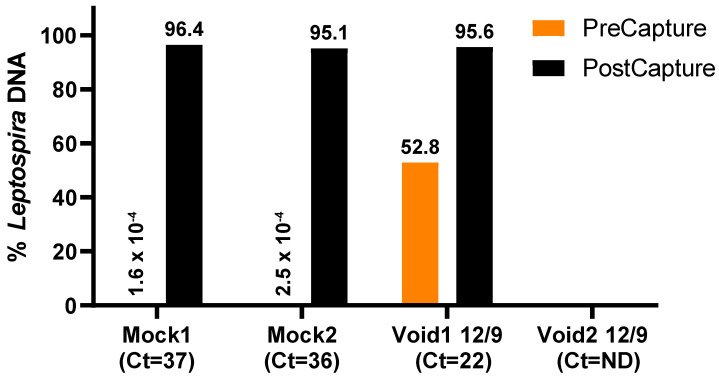
Increase in proportion of *Leptospira* DNA in samples following DNA capture and enrichment. Orange bars indicate the proportion of *Leptospira* DNA before enrichment (not always visible) and black bars indicate the proportion of *Leptospira* DNA after two rounds of enrichment; percentage values are displayed. The *lipL32* PCR Ct values from the original extractions are noted in parentheses. ND indicates not determined.

Of the four validation samples—Mock1, Mock2, Void1 12/9, and Void2 12/9—all three PCR-positive samples (Table 1) revealed significant increases in the proportion of *Leptospira* DNA after two rounds of DNA capture and enrichment, whereas no *Leptospira* DNA was detected in the PCR negative sample either before or after enrichment (Void2 12/9; Figure 1). Starting concentrations of *Leptospira* DNA in the Mock1 and Mock2 sample libraries were estimated to be well below 1%, whereas Void1 12/9 was 52.8%. After two rounds of enrichment, all three of these samples contained >95% *Leptospira* DNA (Figure 1 and Table 2). The enrichments for DCP009, DCP017, and DCP041 contained 79.4–96.2% *Leptospira* DNA in the final enriched libraries and, when mapped against the closed genomes obtained from their corresponding isolates, the breadth of coverage ranged from 89.4–99.9% (Table 2).

#### 3.1.3. Phylogenetic Analysis

**Figure 2:**
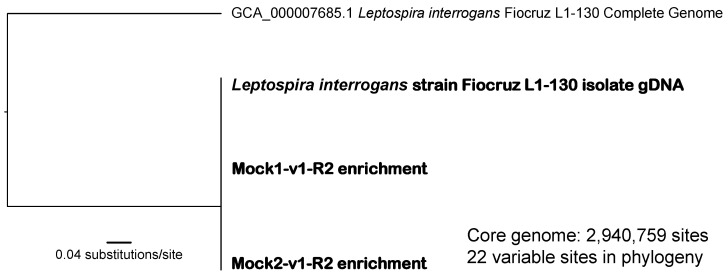
Midpoint rooted maximum likelihood phylogeny including the published complete genome of *Leptospira interrogans* serovar Copenhageni strain Fiocruz L1-130 with one isolate genome generated from a commercially purchased gDNA stock of this strain and two genomes enriched from complex samples spiked with the same gDNA. The phylogeny was constructed using the concatenated alignment of 22 SNPs identified from a core genome of 2,940,759 nucleotide positions shared among these genomes.

Our genomic analyses revealed an average breadth of coverage of 86.9% and 78.2%, respectively, across the twice enriched and non-pooled Mock1 and Mock2 genomes when mapped against the L1-130 isolate genome (L1-130_assembly.fasta). Phylogenetic analysis of these enriched mock genomes together with other publicly available *L. interrogans* genomes facilitated accurate identification of the inoculated gDNA from strain *L. interrogans* serovar Copenhageni strain Fiocruz L1-130 and revealed no SNPs among them (Figure 2 and Figure S1). We did identify 22 SNPs differentiating the complete L1-130 genome available from GenBank (GenBank accession# GCA_000007685.1) from our sequenced L1-130 gDNA (L1-130_assembly.fasta) that was spiked into the Mock samples; these comparisons included >2.9 million shared nucleotide positions (Figure 2).

**Figure 3:**
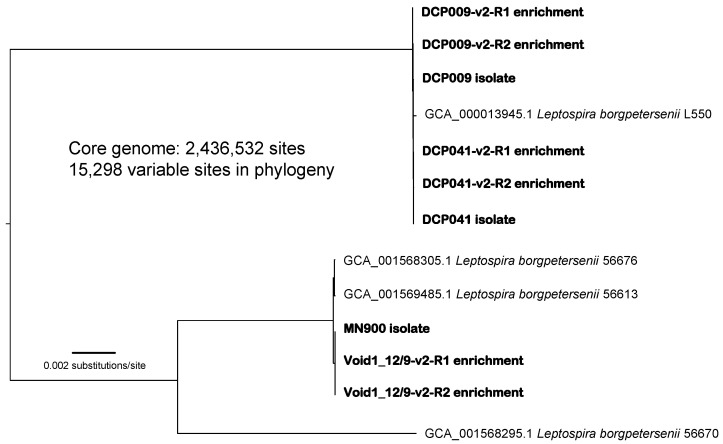
Midpoint rooted maximum likelihood phylogeny including isolate and enriched genomes generated from complex bovine samples Void1 12/9 (MN900), DCP009, and DCP041. This phylogeny was constructed using the concatenated alignment of 15,298 SNPs identified from a core genome of 2,436,532 nucleotide positions shared among these genomes.

Phylogenetic analysis of genomes generated using the v2 capture probes and complex bovine samples Void1 12/9, DCP009, and DCP041 after both one (R1) and two (R2) rounds of enrichment grouped them with the respective genomes generated from the *L. borgpetersenii* isolates obtained from those same complex samples (Figure 3 and Figure S2; note that isolate MN900 was obtained from sample Void1 12/9) and breadth of coverage ranged from 85.7–99.9% (Table 2). No SNPs were identified among the two Void1 12/9 enrichments and the MN900 isolate genome when comparing >2.4 million shared nucleotide positions (Figure 3).

Four putative SNPs were identified between the DCP041 isolate genome and the genomes generated from the two enrichments of the complex sample that yielded this isolate (also named DCP041). When these putative SNPs were manually viewed in Tablet, using genomic coordinates provided in the NASP “bestSNP” matrix output, they were determined to be the result of true SNPs present in the isolate genome that were also present in the enriched genomes but filtered out by NASP because they did not meet the stringent SNP threshold set forth in the analysis (proportion of 0.9 and >10x coverage: see above). Sixteen putative SNPs also were identified among the DCP009 isolate genome and the genomes generated from the two enrichments of the complex sample that yielded this isolate (also named DCP009); all were visualized in Tablet and nine were determined to be analysis artifacts also due to the SNP threshold described above (i.e., not real), whereas seven true SNPs were identified and shared among the two enriched genomes but absent in the isolate genome. The Void1 12/9 enriched genomes and MN900 isolate genome grouped together within a clade that contained other serovar Tarassovi isolates, whereas the DCP009 and DCP041 enriched and isolate genomes grouped within a clade that contained other serovar Hardjo-bovis genomes (Figure S2).

#### 3.1.4. Direct Whole-Genome Comparisons among Paired Isolates and Enrichments

For our first direct comparison, we analyzed the MN900 closed genome (MN900_closed.fasta) along with the Void1 12/9-v2-R1, Void1 12/9-v2-R2, and Void1 12/9-v1-R2 enriched genomes. This comparison encompassed 2,713,814 shared nucleotide positions and revealed no SNPs among them. We then analyzed the DCP041 closed genome (DCP041_closed.fasta) along with the DCP041-v2-R1 and DCP041-v2-R2 enriched genomes. This comparison encompassed 3,036,988 shared nucleotide positions and identified four SNPs, which were all shared between the two enrichments but differentiated them from the isolate genome. This is not surprising given that the isolate genome was derived from a cultured isolate obtained from a urine void that was collected nearly three months after the urine void was used to generate the enrichments (see above).

The direct comparison of the DCP009 closed genome (DCP009_closed.fasta) along with the DCP009-v2-R1 and DCP009-v2-R2 enriched genomes encompassed 3,105,399 shared nucleotide positions and identified ten SNPs shared in the two enriched genomes that differentiated them from the isolate genome. Like sample DCP041, the cultured isolate was obtained from a urine void collected nearly two months after the urine void that was used to generate the enrichments (see above). We also observed 42 additional SNPs in the DCP009-v1-R2 genome when compared to the isolate that were not called in the DCP009-v2-R1 genome. Upon visual examination of the SNPs using a Tablet, it was revealed that these SNPs were an artifact of a low-level coinfection with *L. santarosai* that became a more dominant portion in the DNA library after the second round of enrichment. We then investigated whether these SNPs were also present in the DCP009-v2-R1 genome but at a low enough proportion to evade discovery. Indeed, the contaminating *L. santarosai* reads were also present in the DCP009-v2-R1 genome, but in a much lower proportion than the dominant *L. borgpetersenii* genotype.

We analyzed the inoculated *L. interrogans* serovar Copenhageni strain Fiocruz L1-130 genome (L1-130_assembly.fasta) together with the Mock1-v1-R2 and Mock2-v1-R2 enriched genomes. This comparison included 2,919,473 shared nucleotide positions and revealed six SNPs that were shared in the two enriched genomes and differentiated them from the inoculated genome. Because the gDNA among these comparisons was the same, the presence of these SNPs was likely not due to any natural phenomenon. Upon visualization of these SNPs in Tablet, we discovered that two of them were located on the same sequencing reads. We conducted BLAST analysis of those reads using NCBI blastn and it was revealed that they were a perfect match to *E. coli*, which was present in both human urine DNA samples used to generate Mock1 and Mock2. The remaining four SNPs were also in close proximity to each other and on the same sequencing reads. We applied NCBI blastn to these reads and they were a perfect match to the inoculating *L. interrogans* strain, which was unexpected. We believe that these SNPs are the result of reads from a gene duplication being mapped to the wrong coordinates on the genome (see below).

Finally, our DNA capture and enrichment of soil sample 16^S^-27 revealed that we were unable to capture a significant proportion of *Leptospira* reads (<2% [Table 2]) and that those reads did not match the *L. sanjuanensis* isolates from this same sample (LGVF01 and LGVF02) [30], or any other *Leptospira* isolates, either pathogenic or saprophytic, previously obtained from this sample [26]. That said, the extracted *secY* read pileup contained sequences that shared 100% identity with *secY* sequences generated from other soil samples collected from this same site in a previous study [26].

### 3.2. Unknown Set

#### 3.2.1. Species Identification and Determination of Mixtures

The full-length *secY* gene (1383 bp) was extracted from assemblies for all enrichment iterations for sample KY74, whereas 1383 bp and 707 bp were extracted from PCRpos02-v1-R2 and PCRpos02-v1-R2-P, respectively; 1382 bp and 532 bp were extracted from PCRpos05-v1-R2 and PCRpos05-v1-R2-P, respectively; and 895 bp was extracted from KYcalf-v2-R2. As mentioned above, the reduced size was likely caused by incomplete capture of the *secY* gene for certain samples during enrichment. Species identifications were *L. borgpetersenii* for KY74 and KYcalf, *L. interrogans* for PCRpos02, and *L. kirschneri* for PCRpos05 (Table 2). One hundred percent sequence identity to each species was observed with one exception: for sample PCRpos05, *L. kirschneri* strain I-7 was the closest match with 99.71–99.81% sequence identity (range of values due to different *secY* consensus sequence lengths extracted from PCRpos05-v1-R2 and PCRpos05-v1-R2-P). Mixtures of *L. borgpetersenii* and *L. interrogans* were observed in KYcalf-v2-R2 in similar proportions. Sample WI878 did not contain a high proportion of post-enrichment reads that were assigned to *Leptospira* (<3%), suggesting *Leptospira* DNA was not present in the original sample. We attempted to enrich this sample with both versions of the probes (v1 and v2; Table 2) to explore the possibility that the more comprehensive probe set (v2) would improve enrichment success for this sample, but no noteworthy difference was observed.

#### 3.2.2. Enrichment Results

For unknown human samples PCRpos02 and PCRpos05 we also estimated starting concentrations of *Leptospira* DNA in the unenriched libraries using LeptoBait qPCR. Based on those analyses, PCRpos02 contained 89.9% and PCRpos05 contained 79.6% *Leptospira* DNA after two rounds of DNA capture and enrichment; starting concentrations for both were estimated to be below 1% (Figure S3).

#### 3.2.3. Phylogenetic Analysis

**Figure 4:**
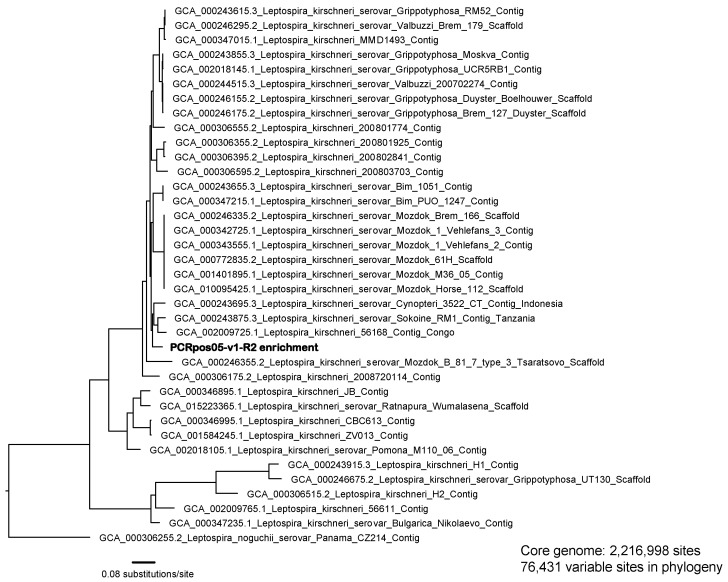
Maximum likelihood phylogeny rooted with *Leptospira noguchii* strain CZ214 and including an enriched genome from human sample PCRpos05 and other *L. kirschneri* genomes (n = 35) downloaded from GenBank. Accession numbers for all GenBank genomes are included in the annotations. The phylogeny was constructed using the concatenated alignment of 76,431 SNPs identified from a core genome of 2,216,998 shared nucleotide positions.

Phylogenetic analysis revealed that unknown human sample PCRpos02 was infected with a *L. interrogans* strain that is most similar to other isolates belonging to the serovar Copenhageni clade (Figure S4). The L1-130 complete genome (L1-130_closed.fasta) was used as the reference for discovery of the SNPs used to construct this phylogeny and the core genome for this analysis encompassed >2.4 million shared nucleotide positions. Unknown human sample PCRpos05 was confirmed to harbor a *L. kirschneri* strain that is notably different from other available *L. kirschneri* genomes included in the phylogeny, with the closest matches belonging to isolates obtained from Africa and Indonesia (Figure 4). Our assembly of *Leptospira kirschneri* strain RedPanda1 (RedPanda1_assembly.fasta) was used as the reference for the discovery of the SNPs used to construct this phylogeny and the core genome for this analysis encompassed >2.2 million shared nucleotide positions.

Our analysis of three bovine samples wherein the infecting *Leptospira* lineages were undetermined demonstrated the power of this approach to (1) identify unknown *Leptospira* genotypes infecting bovines in the continental US (sample KY74), (2) identify and assign species identification to mixtures of infecting strains (sample KYcalf), and (3) confirm the absence of pathogenic *Leptospira* DNA in a suspected leptospirosis case (sample WI878). Both sample enrichments for the Kentucky bovines were highly successful, yielding 78.9–96.0% *Leptospira* DNA in the final enriched libraries (Table 2), and our analysis of those enrichments determined that both were infected with *L. borgpetersenii* and that KYcalf was also infected with *L. interrogans.* Phylogenetic analysis of the KY74 enriched genome, using the MN900 isolate genome as a reference for SNP discovery (MN900_closed.fasta), together with enriched samples Void1 12/9-v2-R2, DCP009-v2-R2, and DCP041-v2-R2 plus 28 additional genomes of *L. borgpetersenii* serovar Hardjo-bovis downloaded from GenBank, revealed that the infecting lineage was, among the strains included in the phylogeny, most closely related to the DCP009 and DCP041 genomes from Puerto Rico (Figure S5). The core genome for this analysis encompassed >2.5 million shared nucleotide positions.

Only trace amounts of *Leptospira* DNA were obtained from the WI878 sample even after two rounds of DNA capture and enrichment and after attempts with both probe designs (v1 and v2). Of the small proportion of reads that were assigned to *Leptospira* using Kraken2 (Table 2), the identity of these reads was identical to the *Leptospira* present in our control DNAs (*L. interrogans* serovar Copenhageni strain Fiocruz L1-130 and/or MN900), which is suggestive of index hopping among samples sharing an Illumina sequencing run. Indeed, the WI878 libraries were sequenced on Illumina runs in which we also sequenced Void1 12/9 (MN900) and the Mock1 and Mock2 enrichments; the latter two were inoculated with the L1-130 strain. This phenomenon is well described elsewhere [49] but nevertheless highlights the need to proceed with caution when detecting low-level sequences of interest. Overall, this result suggests that the clinical symptoms of WI878 were likely not caused by pathogenic *Leptospira* from the P1 clade. However, the positive FAT result strongly suggests the presence of *Leptospira* in this sample. It is possible that the FAT was reacting with a P2 clade *Leptospira* or a saprophytic contaminant. It is also important to note that this sample was delayed in transit for >1 week, which could have led to DNA degradation; indeed, the *lipL32* and *secY* PCRs were also unsuccessful (see above).

### 3.3. DNA Capture and Enrichment Decision Points

Pooling samples after the creation of sequence-ready libraries, but prior to DNA capture and enrichment, is a strategy aimed at reducing overall costs per sample. Our analysis of this approach revealed an overall decrease in the percentage of *Leptospira* reads per sample and, accordingly, a decrease in the breadth and depth of coverage among samples when pooling (Figure S6A). However, only the decrease in the breadth of coverage was statistically significant (*p* = 0.006; Figure S6B). We also observed a wider distribution of sequencing reads (i.e., less uniform) assigned to each pooled sample when compared to the non-pooled counterparts (Table 2). This is likely caused by the variation in the concentration of *Leptospira* DNA within each sample library prior to enrichment, leading to certain samples becoming overrepresented in the sequencing library. Regardless, the breadth of coverage obtained using this pooling approach was similar to non-pooled samples, ranging from 72.6–90.5% (compared to 78.1–86.9% for non-pooled) among the four samples used in this analysis (Figure S6A). This result suggests that pooling sequence-ready libraries prior to enrichment is a viable option for reducing costs associated with DNA capture and enrichment.

In general, two rounds of DNA capture and enrichment were applied to *Leptospira*-positive samples because this has been recently recommended for enriching samples using probe-based DNA capture [50]. The enrichment step is by far the most expensive in the process and so we sought to understand if a second round of enrichment was always necessary, and to also determine what is gained and/or lost during subsequent rounds of enrichment. We observed a general trend of the reduced genomic breadth of coverage when two rounds of enrichment were applied compared to one round of enrichment, but an increase in depth of coverage and in the percentage of *Leptospira* DNA in the final enriched library (Figure S7A). This is in line with what has been described in other systems [50]. In our system, only the reduction in the breadth of coverage after two rounds of DNA capture and enrichment was statistically significant (*p* = 0.0001; Figure S7B). Our results also suggest that two rounds of enrichment are unnecessary when starting concentrations of *Leptospira* within a sample are sufficiently high. For example, bovine samples DCP009, DCP017, and DCP041 displayed high proportions of *Leptospira* DNA within the enriched library after only one round of DNA capture and enrichment (range 79.45–95.21%) (Table 2). Although we did not calculate starting concentrations for those specific samples, our analysis of samples PCRpos02 and PCRpos05 suggest that *lipL32* Ct values ranging from 28.0–32.0 represent starting concentrations of *Leptospira* <1% (Figure S3); *lipL32* Ct values for DCP009, DCP017, and DCP041 were 29.9, 28.3, and 31.4, respectively [32].

Sequencing of the enrichments of samples obtained from bovines in Puerto Rico (DCP009, DCP017, and DCP041) yielded adequate read counts from both rounds of enrichment to conduct this sequencing depth analysis (Table 2). The average percent *Leptospira* for these three samples was 87.1% (range 79.5–95.2%) after one round of enrichment and 95.3% (range 94.4–96.2%) after two rounds. This analysis was aimed at understanding the amount of sequencing depth to target for each enriched sample on an Illumina sequencing platform. We suspect the number of reads allocated to each genome is an important consideration for enriched samples because of the uneven depth of coverage (i.e., peaks and valleys) observed in our enriched assemblies (Figure S8). In general, a statistically significant decrease in the breadth of coverage for both round 1 and round 2 enrichments was observed when 0.5 million paired-end reads were allocated to each sample. Statistical significance for all pairwise comparisons using Tukey’s corrected *p* values is indicated using compact letter display; a method for displaying *p* values whereby pairwise comparisons that share a letter do not reveal statistically significant differences [51] (Figure S9).

As might be expected, higher proportions of *Leptospira* DNA in the final libraries were associated with smaller assemblies (Figure S10). The average genome size of *Leptospira interrogans* ranges from 3.9–4.6 Mb [52] and, therefore, enriched assemblies similar in size likely indicate the presence of fewer contaminating sequences and, thus, are presumably more complete. Our observations suggest that if the goals of analyses are to discover previously uncharacterized *Leptospira* sequences from enrichments using de novo assembly, it is best to maximize the percent *Leptospira* (>95%) in the final enriched library. However, we also performed de novo assembly using only the *Leptospira* sequences classified in Kraken2. For every instance, assemblies were smaller compared to those generated using all the enriched reads (Table S3). We also note that fewer contigs were observed for de novo assemblies generated from samples subjected to only a single round of enrichment (180–517 contigs) compared to two rounds (939–4216 contigs; Table S3).

No statistically significant differences were observed in the breadth of genomic capture obtained between enrichment probe versions when applied separately (or combined) to sample Void1 12/9 (Figure S11) and, as stated above, no SNPs were observed between Void 1 12/9 v1 or v2 enrichments. Because additional probes were added to the v2 design and none were removed, this result is in line with our expectations for the system. This highlights that DNA capture and enrichment probe design is a scalable process that can be updated as needed and as new genomes become available, and that enrichments with previous probe designs will be compatible with enrichments using newer probe designs.

## 4. Discussion

### 4.1. SNP Calling: Potential Sources of Error

On occasion, our genomic analyses identified putative SNPs among samples that contained identical gDNA but were subjected to various iterations of DNA capture and enrichment. Upon careful examination, we identified three phenomena at play that led to these findings. First, enriched genomes were observed to have more dramatic “peaks and valleys” in the depth of coverage when compared to unenriched genomic sequences (Figure S8A,B), which in turn led to areas of lower coverage; an effect that appeared more pronounced after two rounds of DNA capture and enrichment (Figure S8C). We hypothesize that these “peaks and valleys” are due to stochastic differences in probe affinity to certain target sequences over others. Our SNP analysis pipeline (NASP) filters out low-coverage SNPs and, as a result, there were real nucleotide differences in the analyzed genomes (when compared to a reference genome) that were called in the isolate genome due to adequate coverage but filtered out in one or more iterations of the enriched genomes (see results above for DCP009 and DCP041). Second, we observed the mapping of duplicate genomic regions back to the single copy of the reference genome (see results for Mock1 and Mock2). This happens because duplicated regions of the reference genome are removed in NASP (default setting), but not in the query sample(s). When mapping genomes generated from isolates, if SNPs are present in one duplication but not the other, ~50% of the reads at that genomic location would contain SNPs but those SNPs would not be called because they would fall below the SNP threshold, which is typically set at 0.9 (i.e., 90% of the sequencing reads at that position need to contain the SNP for it to be called). However, during DNA capture and enrichment one duplication may have become overrepresented in the library due to the aforementioned stochastic processes and/or differences in probe affinity, thus leading to preferential binding of one-gene duplicates over another. To clarify with an example, if there were two versions of a gene that contained several SNPS between them and only one was captured during enrichment, that version of the gene would be the only mapped representative and, therefore, the SNP calling threshold would be met. We believe that when this occurred, it led to unintentional read proportion biases that resulted in SNP calls at those genomic loci that were inaccurate. Third, we identified SNPs that resulted from contaminating bacterial sequences that remained in the sample post-enrichment and were close enough in sequence identity to the reference genome to be successfully mapped (Mock1 and Mock2). Importantly, the spurious SNPs that resulted from all three phenomena were readily identified by visualization of the SNP locations using the genomic coordinates provided in the “bestSNP” matrix output from NASP in conjunction with the Tablet genome viewer. Moreover, now that these phenomena have been identified and characterized, spurious SNPs could be removed in future analyses by modifying the mapping parameters in NASP. Importantly, in these data sets we did not observe evidence of the incorporation of SNPs during the PCR amplification steps of the enrichment process. That said, we acknowledge this is a possibility and should always be considered when conducting high-resolution SNP analysis of enriched genomes.

### 4.2. Interpretation of Validated SNPs

We identified four SNPs shared between the enriched genomes from DCP041 and ten SNPs in the enriched genomes from DCP009 that distinguished them from their paired isolate genomes. These SNPs passed all quality filters and thus were determined to be legitimate. This observation meets the biological expectations for these samples because, as described above, the enriched genomes were generated from urine voids collected two to three months prior to the urine sample used to obtain the isolates. As such, we suspect that these SNPs were representative of the natural variation present in the community of leptospires within the host and/or reflective of mutations that accumulated over time. Finally, we identified 22 SNPs differentiating the published genome for *L. interrogans* strain Fiocruz L1-130 (GenBank accession# GCA_000007685.1) and our gDNA aliquot for the same strain acquired from ATCC (see Results). This finding highlights the potential for the accumulation of SNPs during the passage of laboratory-maintained stocks of *Leptospira* spp., an important consideration when analyzing closely related genomes and looking for genomic “matches” [53].

### 4.3. Mixed Infections

During our analyses it was discovered that three bovine samples (DCP009, DCP017, and KYcalf) contained mixtures of pathogenic *Leptospira* species. Interestingly, DCP009 and DCP017 were from the same herd in Puerto Rico and were both coinfected with *L. borgpetersenii* and *L. santarosai*. Isolates were previously [32] obtained and characterized from these two bovines (*L. borgpetersenii* from DCP009 and *L. santarosai* from DCP017) and the possibility of coinfection was not pursued. Current diagnostic and epidemiological practices for leptospirosis might argue that the isolation and genomic characterization of a single infecting species would be the definitive diagnostic and epidemiological endpoint for this investigation, but our results have highlighted a potential flaw in that workflow. Indeed, we analyzed six *Leptospira*-positive bovines from Puerto Rico, Minnesota, and Kentucky, and in 50% of those we identified mixed infections. This finding illustrates the complex leptospirosis disease ecology in bovines and highlights the need for molecular tools capable of identifying and characterizing these mixed infections.

In this study, we identified mixtures of *Leptospira* spp. by extracting reads from the *secY* gene, which were then visualized in Tablet. However, other methods for species identification are also possible; we recently described a workflow for detecting and characterizing mixtures of *Francisella* spp. in enriched samples using species-specific probes [29]. Because our *Leptospira* DNA capture and enrichment probe design was based upon the pan-genome of pathogenic *Leptospira*, probes were included in this array that were specific to each species and, thus, this approach could also be applied to this DNA capture and enrichment system. For the mixture identified in sample DCP009, phylogenetic analysis of the dominant lineage (*L. borgpetersenii*) was not impeded because the second infecting species (*L. santarosai*) was a minor component of the enriched sequencing reads. However, for bovine samples DCP017 and KYcalf the almost equal mixtures led to a conglomerate of reads that could not be easily untangled to conduct the genomic level phylogenetic analyses of each infecting species with the bioinformatic methods applied here; fortunately, untangling those reads was not paramount to the present study. That said, we acknowledge that the ability to perform genomic analysis on all species present within a mixture would be the ideal outcome. To that point, we recently described a method for untangling mixtures using metagenome-assembled genomes (MAGs) in the study describing our similar enrichment system for *Francisella* spp. [29].

### 4.4. Best Practices for Cost Savings, Different Analysis Goals, and Sample Types

**Figure 5:**
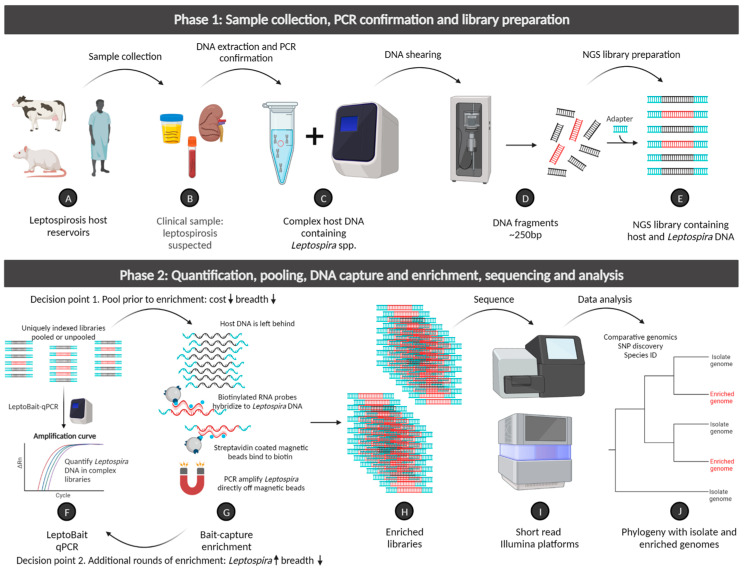
*Leptospira* DNA capture and enrichment workflow outlining input sample types and molecular processes, including library preparation, DNA capture and enrichment, quality control steps, decision points, NGS sequencing, and data analysis. This figure was created with BioRender.com.

DNA capture and enrichment is not inexpensive; we estimate the cost of reagents alone to be ~USD 700/sample, and the laboratory processes involved are not trivial. Indeed, this work requires not only highly skilled laboratorians but also dedicated clean laboratory spaces and equipment to minimize the possibility of sample contamination. As such, we were eager to address the issue of how to make this complex molecular process more affordable and accessible. Several strategies designed to reduce the number of probes used per sample were implemented and assessed; this is because the probes accounted for >70% of our enrichment reagent costs. In this study, we compared genomic data obtained from identical samples enriched separately and also in a pool with three other samples (Figure S6), and our analyses of both suggest that this is a highly effective strategy to significantly reduce probe usage per sample (up to a 75% reduction). To further reduce probe costs, it may also be advantageous to pool samples again after one round of DNA capture and enrichment but prior to a second round; a strategy that we will assess in the future to further refine and reduce the cost of this system. We provide a visual workflow of our pan-pathogenic *Leptospira* DNA capture and enrichment system in Figure 5 to help illustrate this point. Another cost-saving strategy could be to reduce the probe set to just the core genome, which would facilitate genotyping of strains with much higher resolution than single or even multi-gene sequencing. To this point, a DNA capture and enrichment system targeting the core genome of *L. interrogans* with 42,117 probes for the purpose of strain identification within that species was recently described [54]. Due primarily to their fastidious growth requirements, it is also important to consider that the culturing, isolation, propagation, and maintenance of live leptospires for the purposes of whole genome sequencing using traditional approaches is also not a trivial or inexpensive endeavor [55]. Under that lens, the costs associated with DNA capture and enrichment are less prohibitive because all those steps can be circumvented.

The decision to apply one versus two rounds of enrichment should be sample dependent and based upon (1) the starting concentration of *Leptospira* DNA present in the sample, (2) the increase in *Leptospira* DNA after one round of enrichment, and (3) the analysis goals (Figure 5). Our results suggest that when implementing read mapping, similar results can be obtained regardless of whether one round or two rounds of DNA capture and enrichment are applied; we did not obtain <74% *Leptospira* DNA in any of the positive clinical samples analyzed for this study (Table 2). In fact, a single round of enrichment produced a more comprehensive breadth of coverage than two rounds for every comparison (Figure S7) and more complete assemblies, as indicated by fewer contigs when applying de novo assembly to extracted *Leptospira* reads (Table S3). We suspect that during the second round of enrichment probes bias towards sequences that became more abundant after the first round (i.e., common sequences become more common and rare sequences become rarer), resulting in decreased breadth and continuity of coverage. That said, if identifying novel *Leptospira* sequences is the analysis goal, it might be best to conduct de novo assembly on all round two enriched reads (Figure S10) as opposed to de novo assembly on extracted *Leptospira* reads only. In this scenario, the second round of enrichment produced sequencing reads that included fewer contaminating sequences and, thus, could enable the assembly and therefore discovery of non-characterized *Leptospira* sequences. As such, this approach may be more informative for samples that are suspected to contain novel or divergent lineages of *Leptospira* spp. and, thus, are not already well represented in genomic databases. Finally, under the conditions of DNA capture and enrichment applied to samples in this study, we suggest a minimum of 1 million paired-end reads (or >35x coverage) be targeted for each enriched sample (Figure S9).

### 4.5. Other Considerations and Future Perspectives

We observed evidence of index hopping [49] for sample WI878, as indicated by the presence of the few *Leptospira* reads in the sequence associated with this sample assigning to control DNAs (see Section 3) and our overall analyses indicating that this sample was negative for pathogenic *Leptospira* DNA. The library preparation step for the samples described herein, which occurs prior to DNA capture and enrichment, utilized a single indexing scheme that may be prone to index hopping and, thus, the possibility of incorrect assignment of reads to certain samples. To mitigate this potential source of error, we have since modified this procedure to incorporate dual indexing (now available through Agilent), an approach known to reduce or eliminate index hopping [56].

Our attempt to enrich from a single soil sample known to contain pathogenic *Leptospira* [26,30] suggests that this approach may be unsuccessful for highly complex samples with low levels of pathogenic *Leptospira* (such as this bacteria-rich soil from Puerto Rico) because the capture of non-target bacteria may overwhelm the reaction. Perhaps if the pathogenic *Leptospira* load had been higher in this sample and/or the sample had been less complex it would have been successful, a hypothesis that needs to be further explored using this DNA capture and enrichment system. We also note that only 62.2% of the sequencing reads for this sample were classified by Kraken2, which may be suggestive of deficiencies in the database. It is certainly possible that some of the unclassified reads represent non-pathogenic *Leptospira* reads that are currently unrepresented in the Kraken2 database. In line with this, our culturing attempts from this same soil sample in a previous study [26] suggested that it also had an abundance of saprophytic *Leptospira* spp. present in it.

Although we present this DNA capture and enrichment method here with a focus on blood and urine samples from humans and bovines, we acknowledge the existence of other sample types that could benefit from this technique. Indeed, pathogenic *Leptospira* spp. can be found in many different mammal species and are known to colonize a variety of host organs (e.g., kidney, liver) and tissues, including the genital tract of livestock animals [57]. We trust this technique will work equally well on other host sample types because sample complexity (i.e., primarily just a combination of *Leptospira* spp. and host DNA) would be similar to blood or urine.

## 5. Conclusions

Our pan-pathogenic *Leptospira* DNA capture and enrichment system successfully captured, enriched, and produced high-quality *Leptospira* genomic data from complex human and animal samples that had only trace amounts of starting *Leptospira* spp. DNA, with the resulting data enabling high fidelity genomic level phylogenetic comparisons together with genomes generated from isolates. We validated this system using lab-generated controls and complex clinical samples from bovines that contained *Leptospira* spp. already characterized by traditional culturing methods. In addition, we used this DNA capture and enrichment system to characterize human clinical samples (blood and urine), as well as bovine urine samples infected with unknown lineages of pathogenic *Leptospira*. We detected and assigned species identifications to previously unidentified mixed infections in three bovines, and we determined that pooling DNA samples prior to enrichment can be an effective strategy to reduce costs associated with DNA capture and enrichment. We also determined that two rounds of enrichment oftentimes result in a very high percentage of *Leptospira* DNA in the final enriched libraries, but that one round may produce adequate genomic coverage depending on study goals. Most importantly, we have shown that culture-independent DNA capture and enrichment is an amenable and powerful molecular tool that can be applied to leptospirosis genomic research, which will greatly increase the diversity of sample types and the number of samples overall for which genomic information can be obtained.

## Figures and Tables

**Table 1:** Complex samples were enriched in this study and the analyses were applied to each (indicated with an x). The host is indicated as well as sample type and *lipL32* PCR status. Shaded cells indicate validation samples.

Sample ID	Host	Sample Type	*lipL32* PCR	Comparative Isolate?	Sample Set	Analyses				
						Post Capture % Increase	Number of Enrichments	Pooling	Avg. Seq. Depth	De Novo Assembly
Mock1	Human *	Urine	Positive	L1-130	Validation	x		x		x
Mock2	Human *	Urine	Positive	L1-130	Validation	x		x		x
Void1 12/9	Bovine	Urine	Positive	MN900	Validation	x	x			
Void2 12/9	Bovine	Urine	Undetermined	MN900	Validation	x				
DCP009	Bovine	Urine	Positive	DCP009	Validation		x		x	x
DCP017	Bovine	Urine	Positive	DCP017	Validation		x		x	
DCP041	Bovine	Urine	Positive	DCP041	Validation		x		x	x
16^S^-27	Environment	Soil	Positive	LGVF02	Validation					
PCRpos02	Human	Blood	Positive	No	Unknown	x		x		x
PCRpos05	Human	Urine	Positive	No	Unknown	x		x		x
KY74	Bovine	Urine	Positive	No	Unknown		x			x
KYcalf	Bovine	Urine	Positive	No	Unknown					
WI878	Bovine	Urine	Undetermined	No	Unknown					

* DNA extracted from a human urine sample spiked with *Leptospira* DNA (see text).

**Table 2:** Sequencing results for all iterations of DNA capture and enrichment, including average breadth and depth of coverage when mapped to a reference genome.

Sample ID	Enrichment ID ^a^	Probe Set Version	Rounds of Enrichment	Pooled	Total Sequencing Reads	Percent Classified Reads	Percent *Leptospira* Reads	*secY* Consensus Sequence ID (Accession)	Reference Genome Used for Analyses	Average Breadth (>3x)	Average Sequencing Depth (x)
Mock1	Mock1-v1-R2	v1	2	No	1,766,654	99.6	96.4	*L. interrogans* (AE016823.1)	L1-130_assembly.fasta	86.9	54.3
Mock1	Mock1-v1-R2-P	v1	2	Yes	5,057,074	99.3	91.4	*L. interrogans* (AE016823.1)	L1-130_assembly.fasta	90.5	212.4
Mock2	Mock2-v1-R2	v1	2	No	1,194,836	99.6	95.1	*L. interrogans* (AE016823.1)	L1-130_assembly.fasta	78.2	35.6
Mock2	Mock2-v1-R2-P	v1	2	Yes	686,126	98.2	79.3	*L. interrogans* (AE016823.1)	L1-130_assembly.fasta	72.6	22.4
Void1 12/9	Void1129-v1-R2	v1	2	No	1,394,238	97.2	96.0	*L. borgpetersenii* (CP084914.1)	MN900_closed.fasta	86.3	48.5
Void1 12/9	Void1129-v2-R1	v2	1	No	6,399,124	95.7	93.4	*L. borgpetersenii* (CP084914.1)	MN900_closed.fasta	98.5	216.1
Void1 12/9	Void1129-v2-R2	v2	2	No	1,657,708	96.7	95.6	*L. borgpetersenii* (CP084914.1)	MN900_closed.fasta	85.7	57.6
DCP009	DCP009-v2-R1	v2	1	No	9,328,054	96.6	95.2	*L. borgpetersenii* (CP033440.1)	DCP009_closed.fasta	99.8	333.4
DCP009	DCP009-v2-R2	v2	2	No	2,507,792	97.0	96.2	*L. borgpetersenii* (CP033440.1)	DCP009_closed.fasta	90.3	87.4
DCP017	DCP017-v2-R1	v2	1	No	3,378,920	85.5	79.5	*L. santarosai* (CP097245.1)	DCP017_closed.fasta	98.4	92.0
DCP017	DCP017-v2-R2	v2	2	No	2,213,726	95.5	94.4	*L. borgpetersenii* (CP033440.1)	DCP017_closed.fasta	89.4	70.0
DCP041	DCP041-v2-R1	v2	1	No	4,420,192	91.1	86.6	*L. borgpetersenii* (CP033440.1)	DCP041_closed.fasta	99.9	145.4
DCP041	DCP041-v2-R2	v2	2	No	1,539,874	96.5	95.3	*L. borgpetersenii* (CP033440.1)	DCP041_closed.fasta	91.7	54.3
KY74	KY74-v2-R1	v2	1	No	6,964,590	95.6	93.74	*L. borgpetersenii* (CP033440.1)	MN900_closed.fasta	93.1	222.1
KY74	KY74-v2-R2	v2	2	No	2,306,432	96.9	96.0	*L. borgpetersenii* (CP033440.1)	MN900_closed.fasta	82.9	72.2
KYcalf	KYcalf-v2-R2	v2	2	No	3,190,066	83.7	78.9	*L. borgpetersenii* (CP047516.1)	MN900_closed.fasta	57.59	33.78
PCRpos02	PCRpos02-v1-R2	v1	2	No	1,317,502	99.6	89.7	*L. interrogans* (CP048830.1)	L1-130_closed.fasta	82.1	37.6
PCRpos02	PCRpos02-v1-R2-P	v1	2	Yes	4,633,188	99.3	90.3	*L. interrogans* (CP048830.1)	L1-130_closed.fasta	87.5	182.0
PCRpos05	PCRpos05-v1-R2	v1	2	No	964,960	86.2	79.6	*L. kirschneri* (CP112976.1)	RedPanda1_assembly.fasta	78.1	28.1
PCRpos05	PCRpos05-v1-R2-P	v1	2	Yes	1,692,194	95.3	74.6	*L. kirschneri* (CP112976.1)	RedPanda1_assembly.fasta	82.3	57.3
16^S^-27	16^s^-27-v1-R2	v1	2	No	2,916,802	62.2	1.1	*L. kmetyi* (CP033614.1)	LGVF01_closed.fasta	4.9	0.7
WI878	WI878-v1-R2	v1	2	No	4,048,476	70.9	2.7	na	na	na	na
WI878	WI878-v2-R2	v2	2	No	1,472,710	36.0	1.1	na	na	na	na

^a^ v1: probe set v1; v2: probe set v2; R1: one round of enrichment; R2: two rounds of enrichment; na: not applicable.

## Data Availability

All raw sequencing reads generated from DNA capture and enrichment during this study for twelve samples are available in GenBank under BioProject# PRJNA937758. BioSamples are sequentially assigned from SAMN33419159-SAMN33419164 and SAMN33419166-SAMN33419171. Raw sequencing reads for each iteration of DNA capture and enrichment are available in GenBank’s sequence read archive and are sequentially assigned from SRR23749859-SRR23749881. The raw sequencing reads for our ATCC-acquired gDNA stock of *L. interrogans* serovar Copenhageni strain Fiocruz L1-130 can be accessed under SRR23761063, BioSample SAMN33699499.

## Supplementary Materials

The following are available online at https://www.mdpi.com/article/10.3390/microorganisms11051282/s1, Table S1: List of 482 genomes used in the v1 probe design including GenBank accession numbers. Table S2: List of 502 genomes used in the v2 probe design including GenBank accession numbers. Table S3: De novo assembly statistics. Figure S1: Maximum likelihood phylogeny rooted with *Leptospira noguchii* strain CZ214 and including the published genome of *L. interrogans* serovar Copenhageni strain Fiocruz L1-130, one isolate genome generated from a commercially purchased gDNA stock of this strain (red text), two genomes enriched from complex samples spiked with the same gDNA (red text), and other *L. interrogans* serovar Copenhageni genomes (n = 105) downloaded from GenBank (black text). Accession numbers for all GenBank genomes are included in the annotations. This phylogeny was constructed using the concatenated alignment of 2407 SNPs identified from a core genome of 2,478,393 nucleotide positions shared among these genomes. Figure S2: Maximum likelihood phylogeny rooted with *Leptospira santarosai* strain LT_821 and including isolate and enriched genomes from complex bovine samples Void1 12/9 (MN900), DCP009, and DCP041 (red text), as well as other *L. borgpetersenii* genomes (n = 151) downloaded from GenBank (black text). Accession numbers for all GenBank genomes are included in the annotations. This phylogeny was constructed using the concatenated alignment of 53,382 SNPs identified from a core genome of 2,125,041 nucleotide positions shared among these genomes. Figure S3: Increase in *Leptospira* DNA using DNA capture and enrichment, represented as a percentage of total *Leptospira* DNA. Orange bars indicate *Leptospira* DNA before enrichment (not visible at this scale), whereas black bars indicate *Leptospira* DNA after two rounds of enrichment; percentage values are displayed. The *lipL32* PCR Ct values from the original extractions are noted in parentheses. Figure S4: Maximum likelihood phylogeny rooted with *Leptospira noguchii* strain CZ214 and including two enriched genomes (Mock1 and Mock2) with unknown human sample PCRpos02 and other *L. interrogans* serovar Copenhageni genomes (n = 105) downloaded from GenBank. Accession numbers for all GenBank genomes are included in the annotations. The phylogeny was constructed using the concatenated alignment of 2358 SNPs identified from a core genome of 2,418,937 shared nucleotide positions. Figure S5: Maximum likelihood phylogeny rooted with *Leptospira borgpetersenii* serovar Tarassovi strain MN900 (MN900_closed.fasta) and including enriched genomes from bovine samples MN900 (Void1 12/9), DCP009, DCP041, and KY74, as well as other *L. borgpetersenii* serovar Hardjo-bovis genomes (n = 28) downloaded from GenBank. Accession numbers for all GenBank genomes are included in the annotations. The phylogeny was constructed using the concatenated alignment of 12,759 SNPs identified from a core genome of 2,506,970 shared nucleotide positions. Figure S6: Breadth and depth of coverage estimates and percent *Leptospira* DNA in the final enriched libraries among identical samples subjected to pooling prior to enrichment or enrichment without pooling. (A) Individual comparisons for four samples are plotted to display general trends where solid bars indicate non-pooled samples and hatched bars indicate pooled samples. Comparisons using more than 0.5 million paired-end reads are indicated. The “Percent” label on the Y-axis refers to both *Leptospira* reads (line) and genomic breadth (bars). (B) Paired *t*-tests were used to assess statistical significance pertaining to differences in percent *Leptospira*, as well as breadth and depth of coverage among treatments (non-pooled [U] vs. pooled [P]); asterisks indicate statistical significance, whereas “ns” is not significant. Figure S7: Breadth and depth of coverage estimates and percent *Leptospira* DNA in the final enriched libraries among identical samples subjected to one or two rounds of DNA capture and enrichment. (A) Individual comparisons for five samples are plotted to display general trends where solid bars indicate samples subjected to one round of enrichment and hatched bars indicate two rounds. Comparisons using fewer than 2 million paired-end reads are indicated. The “Percent” label on the Y-axis refers to both *Leptospira* reads and genomic breadth. (B) Paired *t*-tests were used to assess statistical significance pertaining to differences in percent *Leptospira*, as well as breadth and depth of coverage using one vs. two rounds of enrichment; asterisks indicate statistical significance, whereas “ns” is not significant. Figure S8: Read pileup of the same genomic location for (A) sample MN900 unenriched and (B) after one (Void1 12/9-v2-R1) and (C) two (Void1 12/9-v2-R2) rounds of enrichment. We note a general trend of more dynamic “peaks and valleys” as additional rounds of DNA capture and enrichment are applied, as compared to genomes generated from isolates. Panel A is the genome generated from Illumina reads for isolate MN900 obtained from urine Void1 12/9, Panel B is the enriched genome generated from the same urine void but subjected to one round of enrichment, and Panel C is the same urine void subjected to two rounds of DNA capture and enrichment. Visualizations were generated in Tablet [43]. Figure S9: Bar plots displaying breadth and depth of coverage estimates when allocating 0.5–2.5 million paired-end reads to enriched genomes subjected to (A) one or (B) two rounds of DNA capture and enrichment. Pairwise comparisons that share a letter did not reveal statistically significant differences. Figure S10: Smaller de novo assembly size corresponds to higher percentages of *Leptospira* in the final enriched library. The dotted line represents an approximate genome size of pathogenic *Leptospira* and is included as a point of reference. Figure S11: Breadth and depth of genomic coverage among v1 and v2 probe designs for the pan-pathogenic *Leptospira* DNA capture and enrichment system. No statistically significant differences were observed.
