# Supplementary material for: DNA Capture and Enrichment: A Culture-Independent Approach for Characterizing the Genomic Diversity of Pathogenic Leptospira Species
Source: Microorganisms. 2023 May 14;11(5):1282. doi: 10.3390/microorganisms11051282 (PMC10224534; doi:10.3390/microorganisms11051282)
Supplement: Supplementary file 1 [file microorganisms-11-01282-s001.zip › FigureS5.pdf]

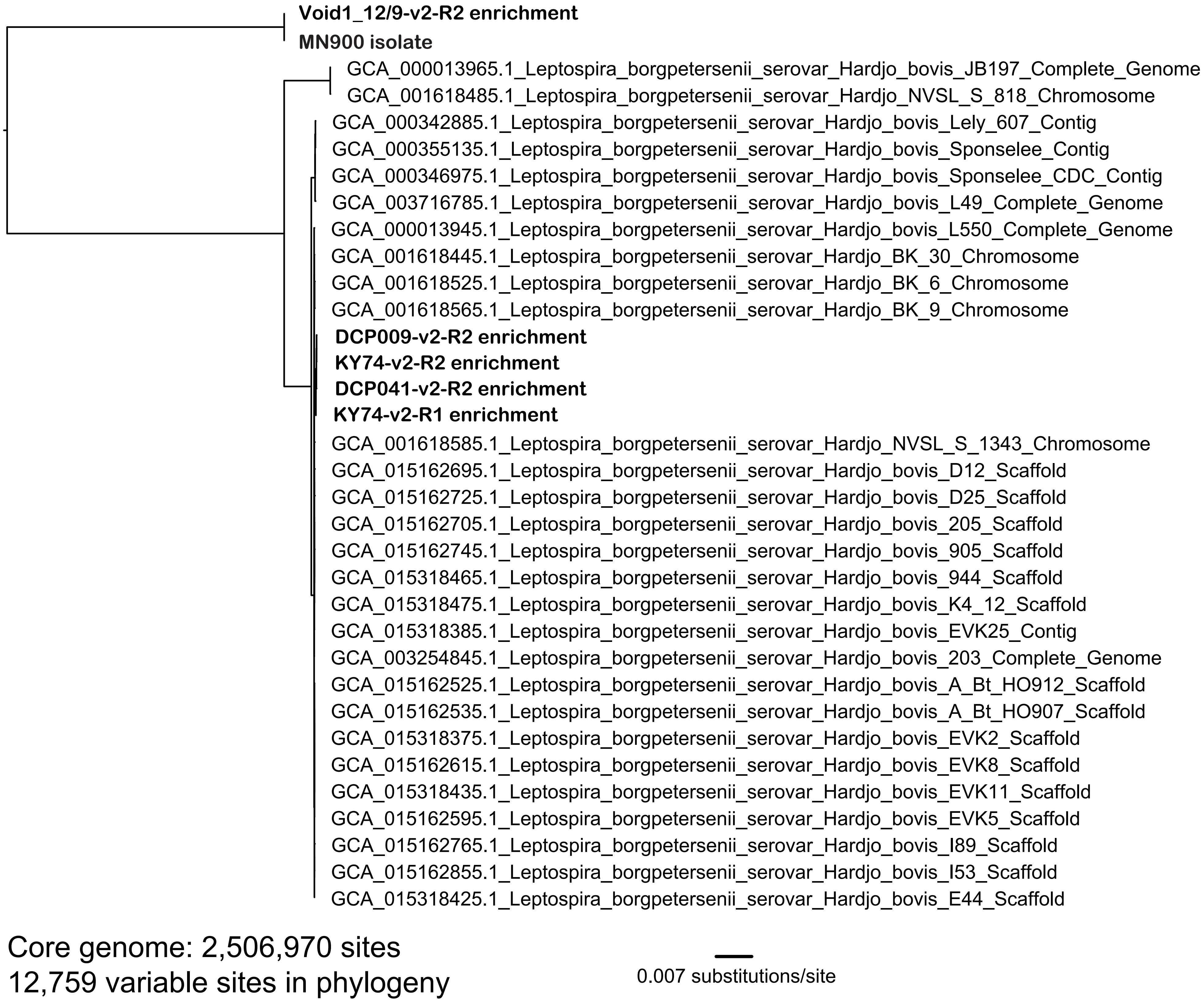

Void1_12/9-v2-R2 enrichment
MN900 isolate
GCA_000013965.1_Leptospira_borgpetersenii_serovar_Hardjo_bovis_JB197_Complete_Genome
GCA_001618485.1_Leptospira_borgpetersenii_serovar_Hardjo_NVSL_S_818_Chromosome
GCA_000342885.1_Leptospira_borgpetersenii_serovar_Hardjo_bovis_Lely_607_Contig
GCA_000355135.1_Leptospira_borgpetersenii_serovar_Hardjo_bovis_Sponselee_Contig
GCA_000346975.1_Leptospira_borgpetersenii_serovar_Hardjo_bovis_Sponselee_CDC_Contig
GCA_003716785.1_Leptospira_borgpetersenii_serovar_Hardjo_bovis_L49_Complete_Genome
GCA_000013945.1_Leptospira_borgpetersenii_serovar_Hardjo_bovis_L550_Complete_Genome
GCA_001618445.1_Leptospira_borgpetersenii_serovar_Hardjo_BK_30_Chromosome
GCA_001618525.1_Leptospira_borgpetersenii_serovar_Hardjo_BK_6_Chromosome
GCA_001618565.1_Leptospira_borgpetersenii_serovar_Hardjo_BK_9_Chromosome
DCP009-v2-R2 enrichment
KY74-v2-R2 enrichment
DCP041-v2-R2 enrichment
KY74-v2-R1 enrichment
GCA_001618585.1_Leptospira_borgpetersenii_serovar_Hardjo_NVSL_S_1343_Chromosome
GCA_015162695.1_Leptospira_borgpetersenii_serovar_Hardjo_bovis_D12_Scaffold
GCA_015162725.1_Leptospira_borgpetersenii_serovar_Hardjo_bovis_D25_Scaffold
GCA_015162705.1_Leptospira_borgpetersenii_serovar_Hardjo_bovis_205_Scaffold
GCA_015162745.1_Leptospira_borgpetersenii_serovar_Hardjo_bovis_905_Scaffold
GCA_015318465.1_Leptospira_borgpetersenii_serovar_Hardjo_bovis_944_Scaffold
GCA_015318475.1_Leptospira_borgpetersenii_serovar_Hardjo_bovis_K4_12_Scaffold
GCA_015318385.1_Leptospira_borgpetersenii_serovar_Hardjo_bovis_EVK25_Contig
GCA_003254845.1_Leptospira_borgpetersenii_serovar_Hardjo_bovis_203_Complete_Genome
GCA_015162525.1_Leptospira_borgpetersenii_serovar_Hardjo_bovis_A_Bt_HO912_Scaffold
GCA_015162535.1_Leptospira_borgpetersenii_serovar_Hardjo_bovis_A_Bt_HO907_Scaffold
GCA_015318375.1_Leptospira_borgpetersenii_serovar_Hardjo_bovis_EVK2_Scaffold
GCA_015162615.1_Leptospira_borgpetersenii_serovar_Hardjo_bovis_EVK8_Scaffold
GCA_015318435.1_Leptospira_borgpetersenii_serovar_Hardjo_bovis_EVK11_Scaffold
GCA_015162595.1_Leptospira_borgpetersenii_serovar_Hardjo_bovis_EVK5_Scaffold
GCA_015162765.1_Leptospira_borgpetersenii_serovar_Hardjo_bovis_I89_Scaffold
GCA_015162855.1_Leptospira_borgpetersenii_serovar_Hardjo_bovis_I53_Scaffold
GCA_015318425.1_Leptospira_borgpetersenii_serovar_Hardjo_bovis_E44_Scaffold
Core genome: 2,506,970 sites
12,759 variable sites in phylogeny
0.007 substitutions/site
