# Supplementary figures and images for: DNA Capture and Enrichment: A Culture-Independent Approach for Characterizing the Genomic Diversity of Pathogenic Leptospira Species
Source: Microorganisms. 2023 May 14;11(5):1282. doi: 10.3390/microorganisms11051282 (PMC10224534; doi:10.3390/microorganisms11051282)

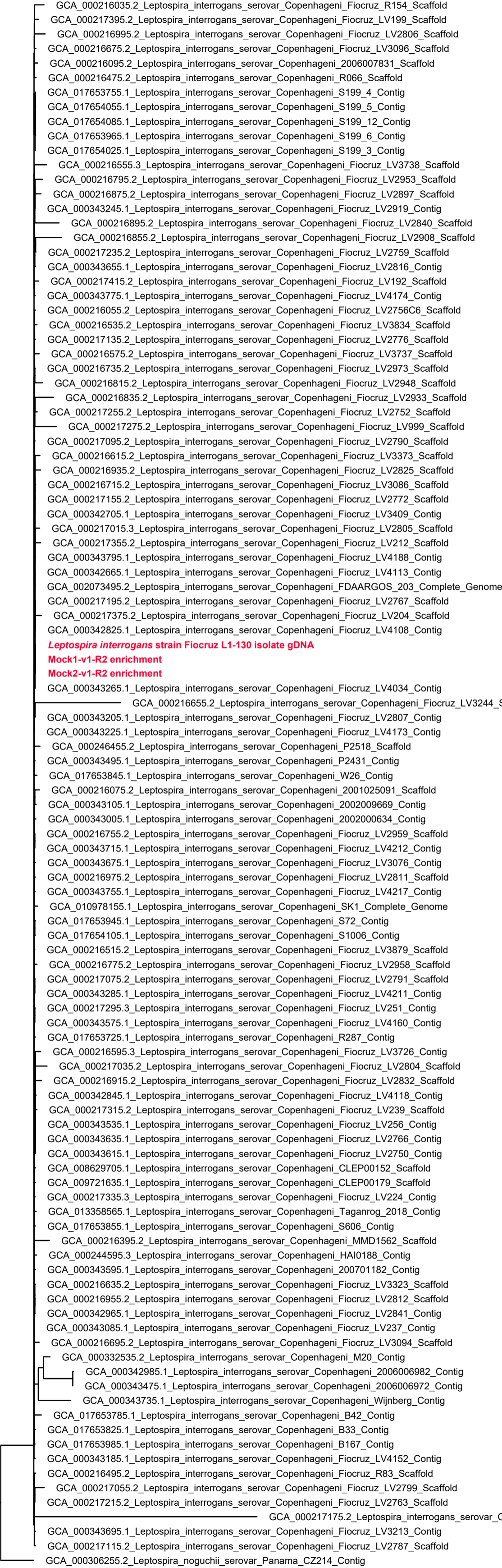

Core genome: 2,478,393 sites  
2,407 variable sites in phylogeny

Supplement: Supplementary file 1 [file microorganisms-11-01282-s001.zip › FigureS1.pdf]

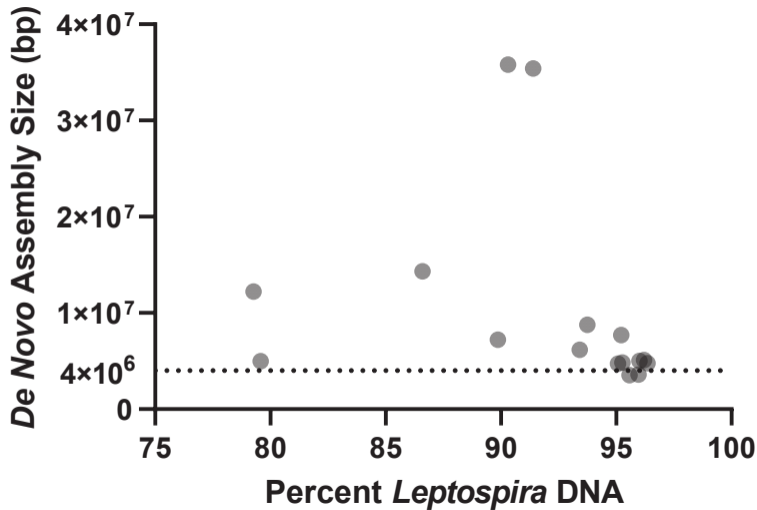

Supplement: Supplementary file 1 [file microorganisms-11-01282-s001.zip › FigureS10.pdf]

## Void1 12/9

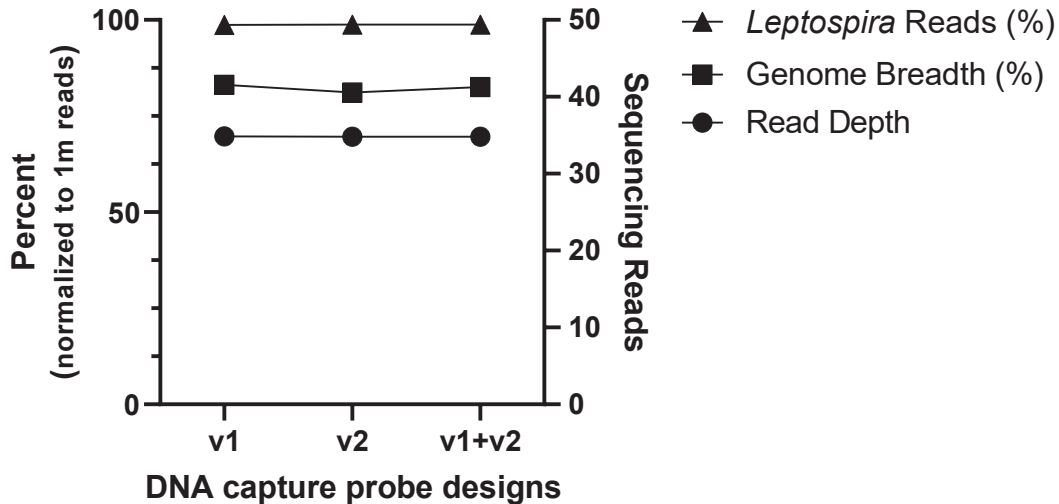

Supplement: Supplementary file 1 [file microorganisms-11-01282-s001.zip › FigureS11.pdf]

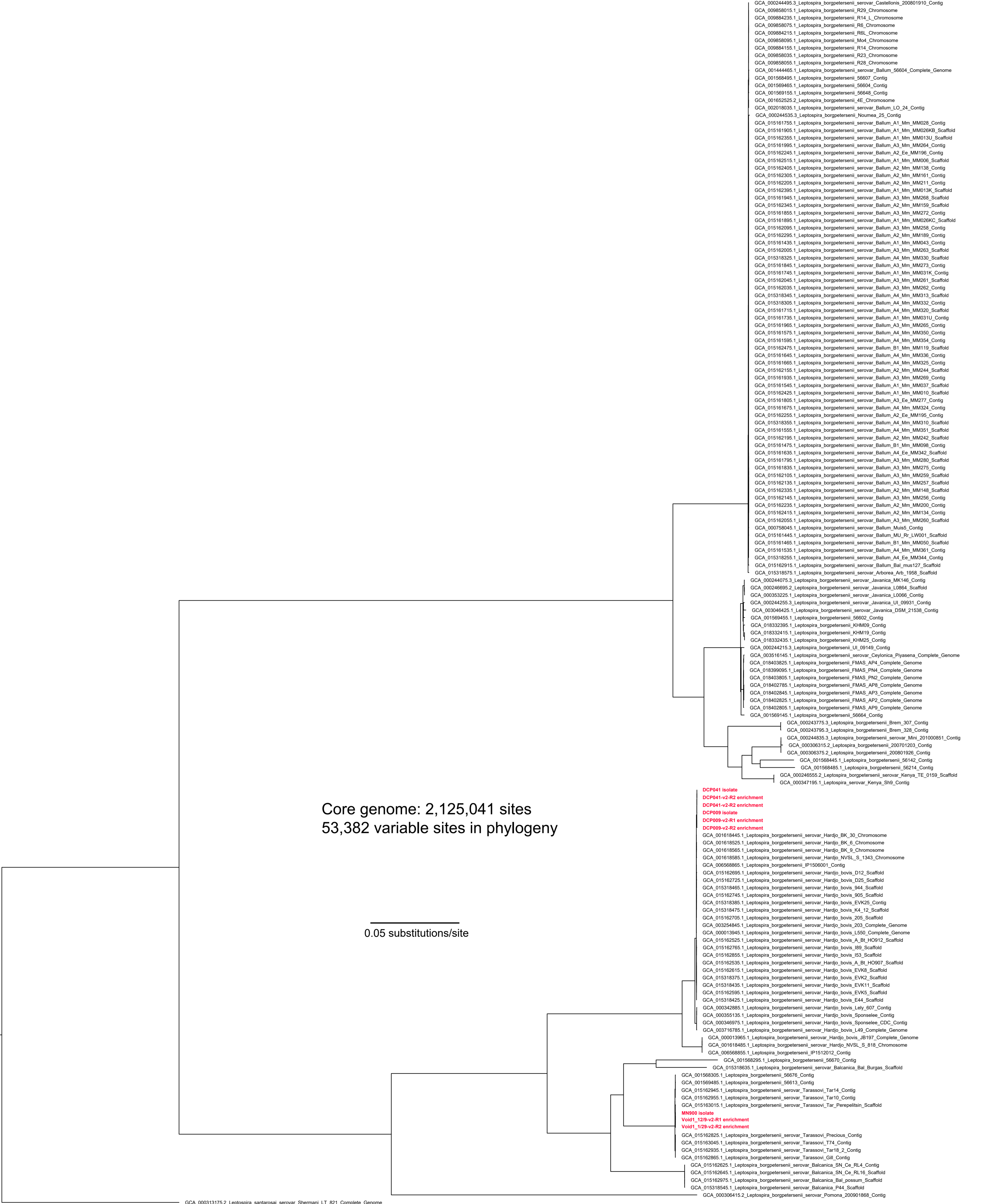

Supplement: Supplementary file 1 [file microorganisms-11-01282-s001.zip › FigureS2.pdf]

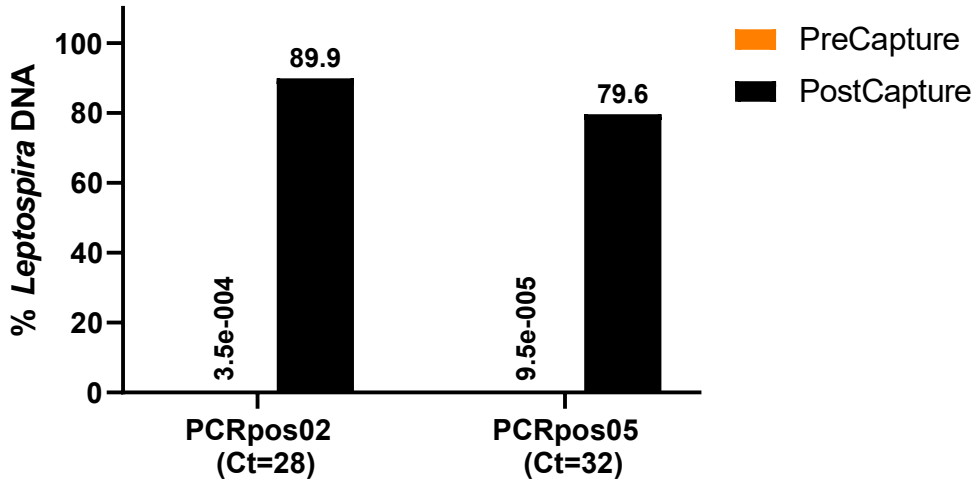

Supplement: Supplementary file 1 [file microorganisms-11-01282-s001.zip › FigureS3.pdf]

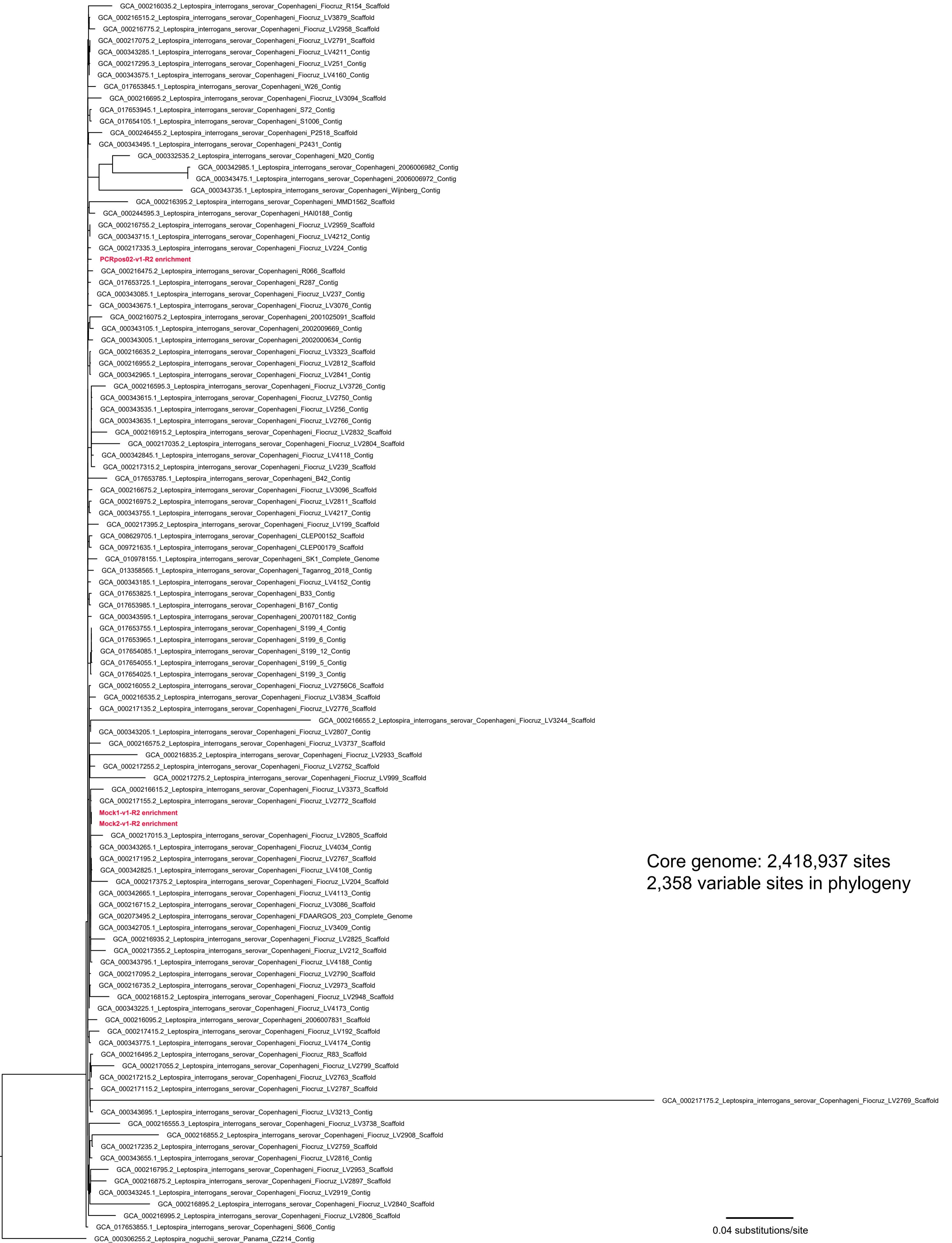

Supplement: Supplementary file 1 [file microorganisms-11-01282-s001.zip › FigureS4.pdf]

A

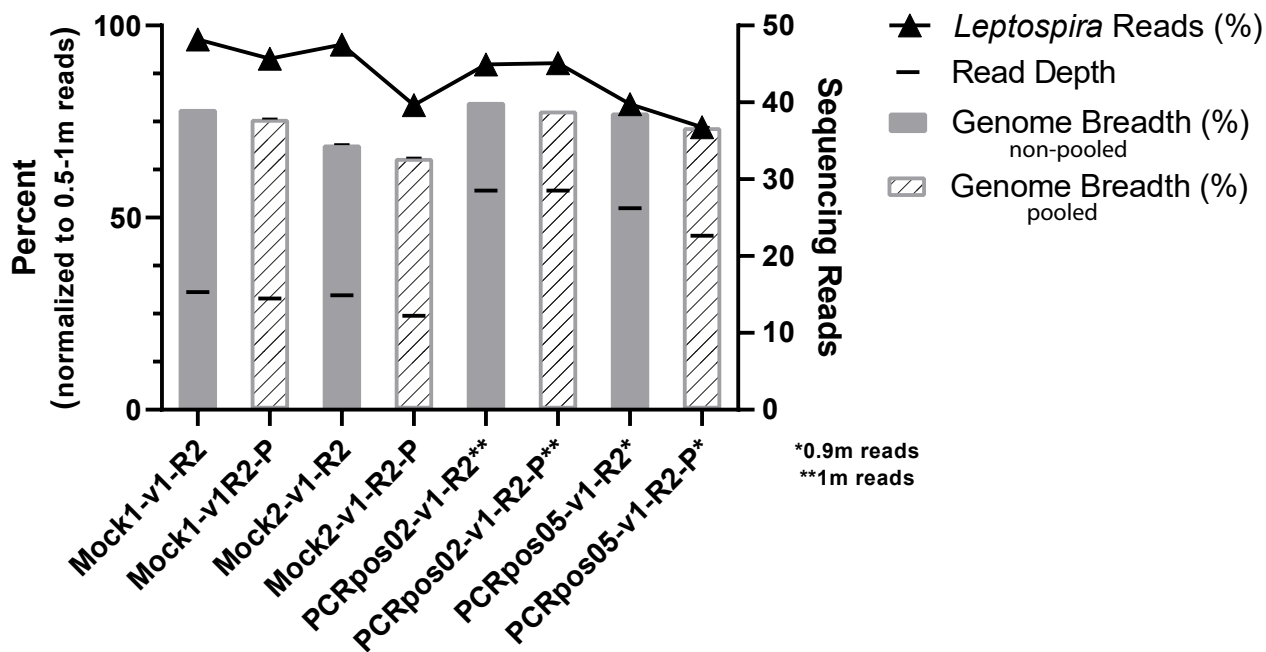

B

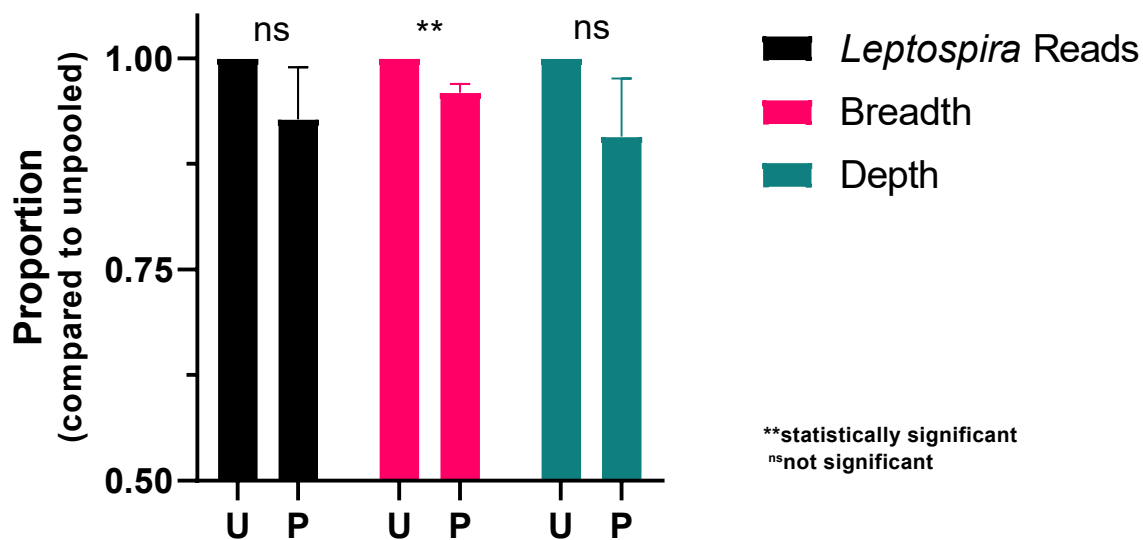

Supplement: Supplementary file 1 [file microorganisms-11-01282-s001.zip › FigureS6.pdf]

A

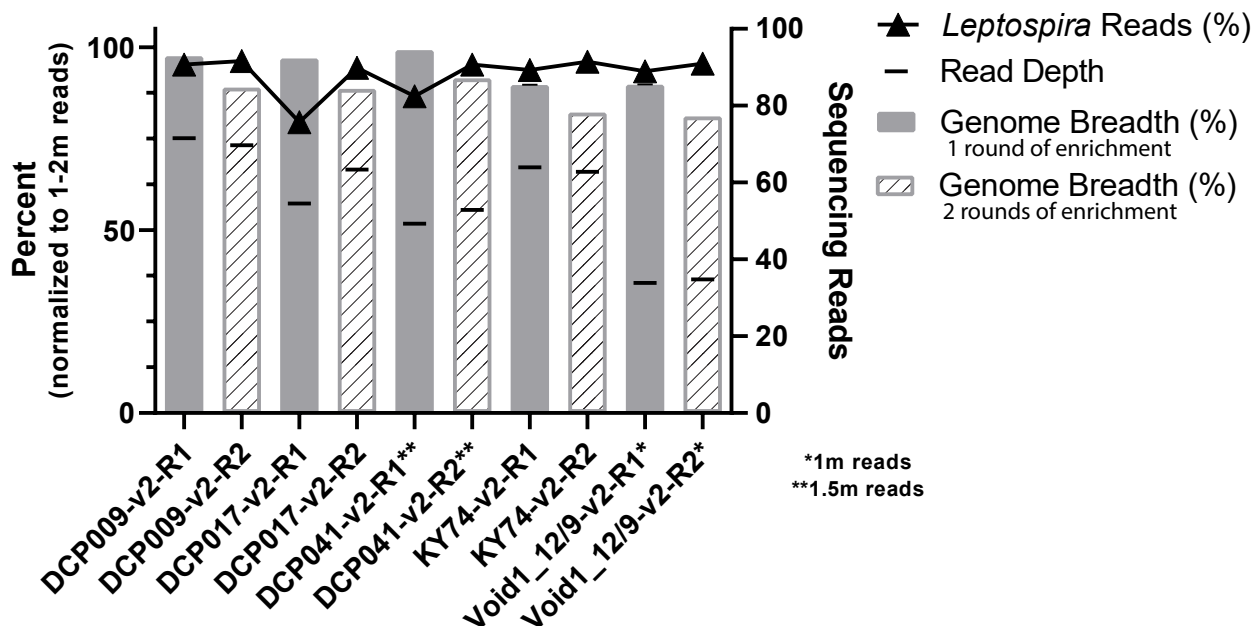

B

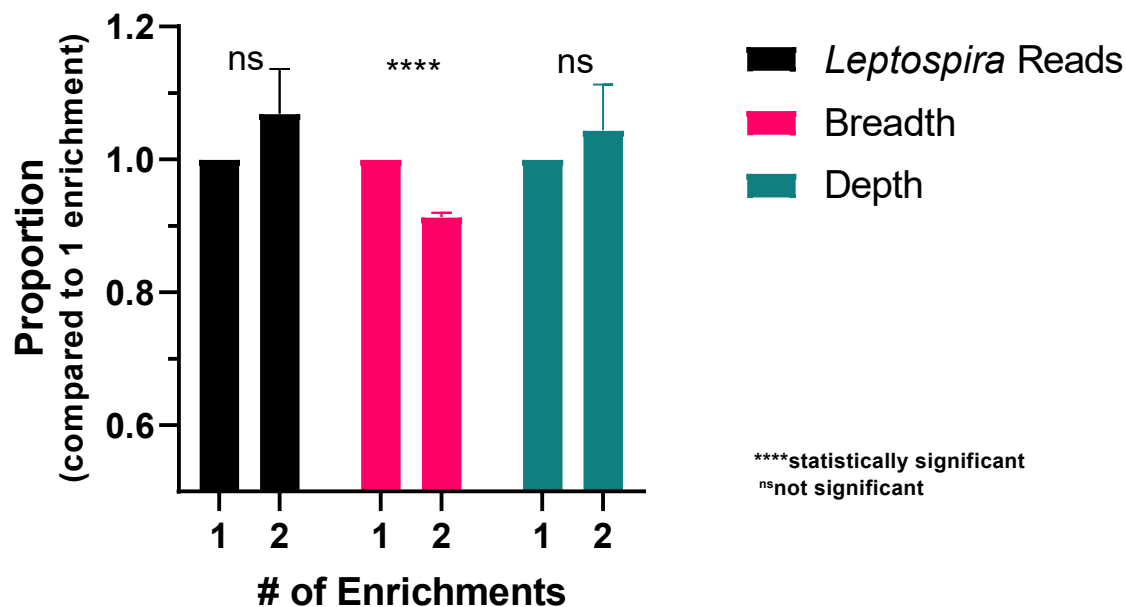

Supplement: Supplementary file 1 [file microorganisms-11-01282-s001.zip › FigureS7.pdf]

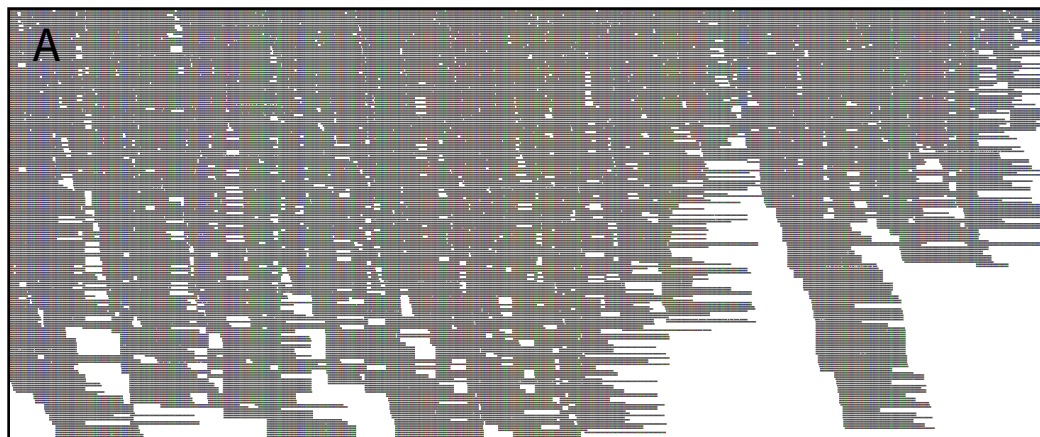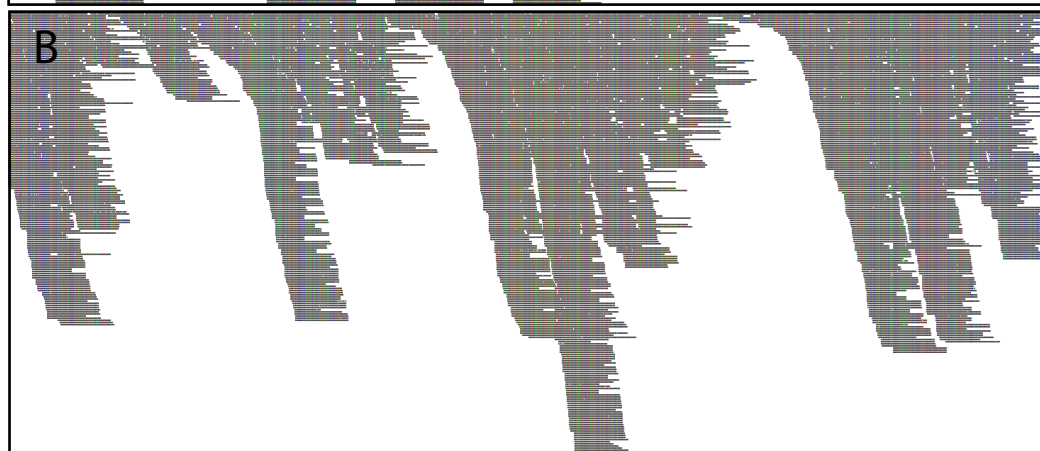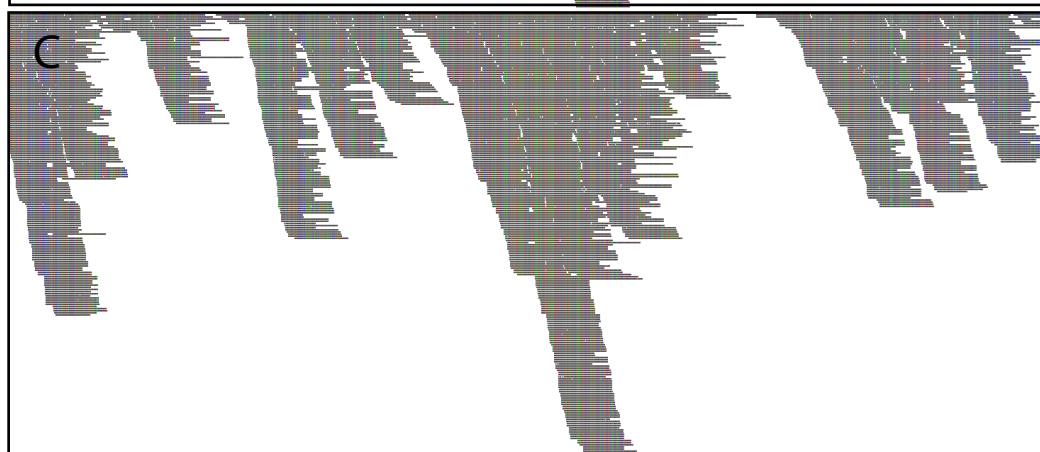

Supplement: Supplementary file 1 [file microorganisms-11-01282-s001.zip › FigureS8.pdf]

A

87.1% *Leptospira*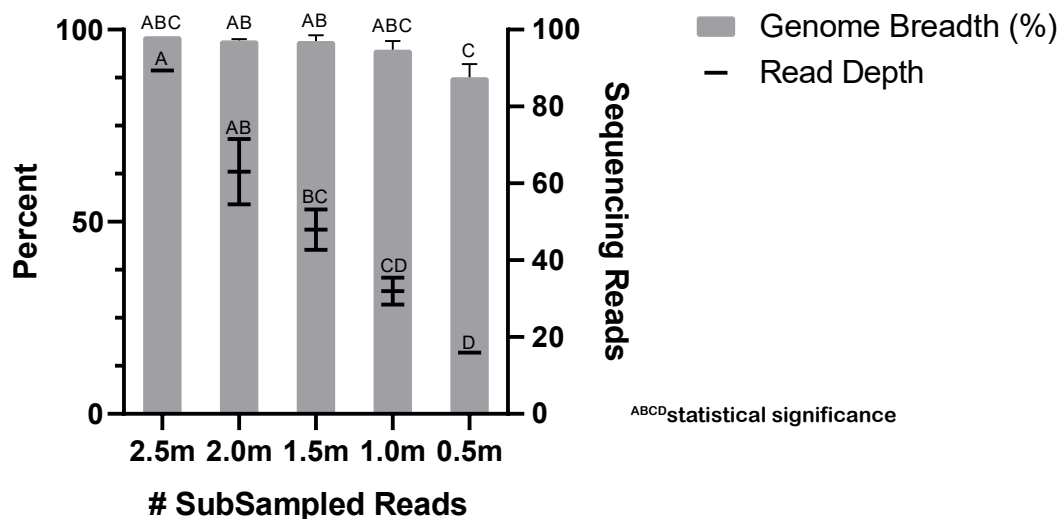

B

95.3% *Leptospira*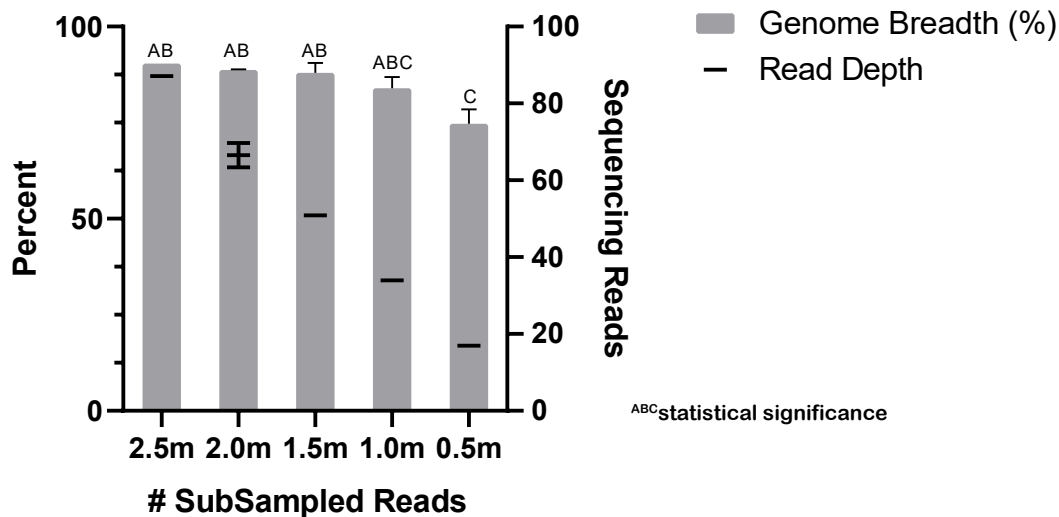

Supplement: Supplementary file 1 [file microorganisms-11-01282-s001.zip › FigureS9.pdf]
